# Testicular endothelial cells are a critical population in the germline stem cell niche

**DOI:** 10.1038/s41467-018-06881-z

**Published:** 2018-10-22

**Authors:** Dong Ha Bhang, Bang-Jin Kim, Byung Gak Kim, Keri Schadler, Kwan-Hyuck Baek, Yong Hee Kim, Wayland Hsiao, Bi-Sen Ding, Shahin Rafii, Mitchell J. Weiss, Stella T. Chou, Thomas F. Kolon, Jill P. Ginsberg, Buom-Yong Ryu, Sandra Ryeom

**Affiliations:** 1 Department of Cancer Biology, Perelman School of Medicine at the University of Pennsylvania, 421 Curie Boulevard, Philadelphia, PA 19104 USA; 2 Department of Molecular and Cellular Biology, Sungkyunkwan University School of Medicine, Suwon, Gyeonggi 440-746, Korea; 3 BK21Plus Program for 21st Century Biomedical Science Leader Development, Sungkyunkwan University School of Medicine, Suwon, Gyeonggi 440-746, Korea; 4 Department of Animal Science and Technology, Chung-Ang University, Ansung, Gyeonggi-Do 456-756, Korea; 5 Ansary Stem Cell Institute, Howard Hughes Medical Institute, Department of Genetic Medicine, Weill Cornell Medical College, 1300 York Avenue, New York, NY 10065 USA; 6 Department of Hematology, St. Jude Children’s Research Hospital, 262 Danny Thomas Place, Memphis, TN 38105 USA; 7 Division of Hematology, The Children’s Hospital of Philadelphia, 3615 Civic Center Boulevard, Philadelphia, PA 19104 USA; 8 Division of Urology, Children’s Hospital of Philadelphia, Department of Surgery (Urology), Perelman School of Medicine at the University of Pennsylvania, 3401 Civic Center Boulevard, Philadelphia, PA 19104 USA; 9 Division of Oncology, Children’s Hospital of Philadelphia, Department of Pediatrics, Perelman School of Medicine at the University of Pennsylvania,, 3501 Civic Center Boulevard, Philadelphia, PA 19104 USA; 10 Department of Pediatrics, The University of Texas MD Anderson Cancer Center, 1515 Holcombe Blvd, Houston, TX 77030 USA

## Abstract

Maintenance of adult tissues depends on stem cell self-renewal in local niches. Spermatogonial stem cells (SSC) are germline adult stem cells necessary for spermatogenesis and fertility. We show that testicular endothelial cells (TECs) are part of the SSC niche producing glial cell line-derived neurotrophic factor (GDNF) and other factors to support human and mouse SSCs in long-term culture. We demonstrate that FGF-2 binding to FGFR1 on TECs activates the calcineurin pathway to produce GDNF. Comparison of the TEC secretome to lung and liver endothelial cells identified 5 factors sufficient for long-term maintenance of human and mouse SSC colonies in feeder-free cultures. Male cancer survivors after chemotherapy are often infertile since SSCs are highly susceptible to cytotoxic injury. Transplantation of TECs alone restores spermatogenesis in mice after chemotherapy-induced depletion of SSCs. Identifying TECs as a niche population necessary for SSC self-renewal may facilitate fertility preservation for prepubertal boys diagnosed with cancer.

## Abstract (web summary)

Self-renewal of spermatogonial stem cells (SSC) is necessary for spermatogenesis and male fertility. Here the authors identify testicular endothelial cells (TECs) as a source of 5 key growth factors for self-renewal and expansion of human and mouse SSCs.

## Introduction

Adult mammalian tissues are maintained by stem cell populations that self-renew in specialized organ-specific niches providing the factors necessary for their maintenance^1,2^. However, for most organs, the niche cells necessary for stem cell self-renewal have not yet been identified. Spermatogonial stem cells (SSCs) are well-characterized adult stem cells necessary for fertility. However, the cellular populations in the SSC niche have not yet been described and although endothelial cells (ECs) in other organs contribute to stem cell niches, a role for testicular endothelial cells (TECs) in the SSC niche has not been examined. Studies have shown that bone marrow ECs are critical in the hematopoietic stem cell (HSC) vascular niche producing stem cell factor, necessary for HSC maintenance and self-renewal in the bone marrow^3–5^. Brain ECs are another example of ECs in a stem cell niche as brain endothelium contributes to neural stem cell maintenance via secretion of vascular endothelial growth factor (VEGF) among other factors^6,7^. It is increasingly evident that endothelium functions in an organ-specific manner to both regulate developmental processes and maintain normal organ homeostasis via production of tissue-specific secretomes^8,9^.

SSCs are an adult stem cell population within the testis that self-renew maintaining productive spermatogenesis in the adult male. Previous studies have identified glial-derived neurotrophic factor (GDNF) as critical for SSC self-renewal with transgenic loss- and gain-of-function mouse models of GDNF confirming the necessity of this factor for the maintenance of SSCs^10^. After the observation that GDNF was necessary for spermatogenesis, culture conditions for mouse SSCs were rapidly developed with the addition of GDNF and other growth factors sufficient to maintain mouse SSCs cultured on embryonic fibroblast feeder cells for months^11^. SSCs harvested from mice and other animals can now be routinely expanded, and although previously published studies have described conditions for culturing human testicular cells^12,13^, expansion of human SSCs for clinical use cannot yet be reproducibly or routinely performed. This roadblock is due in part to our lack of knowledge regarding the identity of the critical SSC niche cells which produce GDNF and other factors. GDNF is expressed by Sertoli cells and peritubuluar myoid (PTM) cells^14^, but there are no definitive studies showing that either of these GDNF-producing populations can support the long-term maintenance and expansion of SSCs. Previous studies suggested that GDNF may be expressed by vascular cells in the testes. GDNF expression was detected by immunohistochemistry in the arterioles and arteries of the testes^15^ and transcriptional analysis of testicular endothelium suggest that TECs could be a source of GDNF^16^. However, the role of TECs in the SSC niche has not yet been investigated. The inability to maintain human SSCs in culture has detrimental consequences on the quality of life for prepubertal boys diagnosed with cancer. SSCs are particularly sensitive to cytotoxic therapies and these patients lack options to obtain mature sperm, and thus many become permanently infertile after completion of cancer treatment.

Recent estimates suggest that 1 in 530 young adults between the ages of 20 and 39 years is a survivor of childhood cancer^17^. While post-pubertal males diagnosed with cancer have fertility preservation options, no options exist for prepubertal boys. In the 1990s, it was demonstrated that spermatogenesis could be restored in mice sterilized after treatment with the chemotherapeutic agent busulfan by injecting germ cells from a syngeneic donor into their seminiferous tubules^18,19^. These results suggested that SSCs might be harvested, before the start of chemotherapy and reintroduced into the testis upon treatment completion. However, testicular biopsies from prepubertal boys contain only a minute number of SSCs and, hence, require expansion in vitro prior to subsequent reinjection.

Here we show that TECs are a key population in the male germline stem cell niche providing necessary growth factors for self-renewal and expansion of human and mouse SSCs in culture. We show that injection of TECs alone is sufficient to restore spermatogenesis in mice after chemotherapy-induced depletion of SSCs and that TECs, but not other organ endothelium, express growth factors that are sufficient for the maintenance of SSCs in culture and include GDNF, fibroblast growth factor-2 (FGF-2), stromal cell-derived factor-1 (SDF-1), macrophage inflammatory protein 2 (MIP-2) and insulin-like growth factor binding protein 2 (IGFBP-2). Our work reveals that both human and mouse SSCs can be cultured long term under feeder-free conditions by the addition of these five factors to the media. Further, our data demonstrate that GDNF expression is specifically driven by FGF-2 binding to FGF receptor 1 (FGFR1) activating the calcineurin (CaN)–nuclear factor of activated T-cells (NFAT) pathway in TECs. We show that dysregulation of CaN–NFAT signaling in ECs can disrupt spermatogenesis and fertility as evidenced in Down syndrome (DS) male mice. We and others have previously shown that EC activation is impaired in DS due to increased expression of chromosome 21-encoded genes that specifically attenuates the CaN–NFAT pathway^20^. A DS mouse model shows defects in SSC self-renewal and/or maintenance and males with DS have significantly reduced sperm counts and are infertile^21,22^. Collectively, our data support the conclusion that the SSC niche is created in part by TECs providing the necessary factors for SSC self-renewal and we identify the CaN–NFAT pathway in TECs as regulating the expression of GDNF, the most critical factor for the maintenance of spermatogenesis.

## Results

### TEC transplantation restores spermatogenesis

**Fig. 1:**
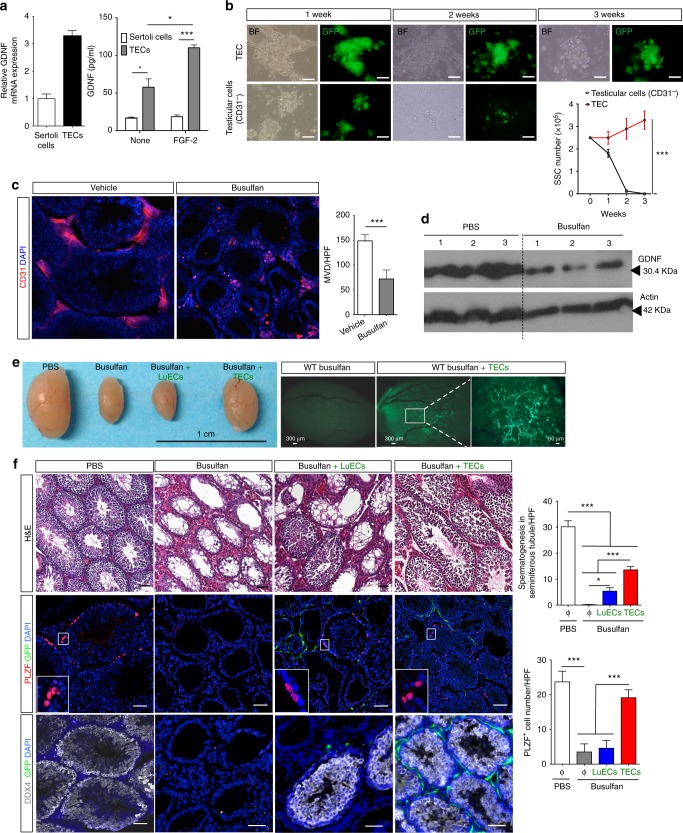
TEC transplantation restores spermatogenesis after busulfan treatment. **a** Quantification of *Gdnf* mRNA in Sertoli cells and TECs, and GDNF levels by ELISA in conditioned media from Sertoli cells or TECs without or with FGF-2 (20 ng ml^−1^) treatment for 3 days. Data are representative of 3 experiments. *P < 0.05, ***P < 0.001, Bars and error bars show the mean ± s.e.m. **b** Murine GFP^+^Thy1.2^+^SSCs were co-cultured with TECs or testicular cells depleted of CD31+ TECs but containing all other cell populations in the testes. After 3 weeks, SSC colonies were absent in CD31-depleted testicular cell co-cultures with Sertoli and PTM cells present, while a significant number of GFP^+^ Thy1.2^+^ SSC colonies were present in co-cultures with TECs alone. Data are representative of two independent experiments. Two-way ANOVA test. **c** Immunofluorescence staining for CD31 (for ECs) on testis sections from wild-type mice 5 weeks after vehicle or busulfan treatment. Microvessel density (MVD) are quantified on the right, *n* = 3–5. **d** Western blot analysis of GDNF expression in whole testis lysates harvested from WT mice 5 weeks after PBS or busulfan treatment. Actin was probed as a loading control and each lane is an individual testis. **e** Whole testes from mice after treatment with PBS, busulfan or busulfan plus transplantation of either mouse LuECs or TECs. Bright-field image: bar = 1 cm. Fluorescent images (bar = 300 μm) show testis from WT mice treated with busulfan with and without transplanted GFP^+^ TECs. **f** Images of testis sections harvested from WT mice after busulfan treatment with and without transplantation of GFP^+^ TECs or GFP^+^ LuECs. Sections were stained with H&E and immunostained for the stem cell markers, PLZF (red) or DDX4 (white) or for GFP (green) to detect transplanted ECs. Quantification of seminiferous tubules showing spermatogenesis per high-powered field (HPF) and PLZF^+^ cells per HPF is shown on right, *n* = 3–4. Immunofluorescence images of testis sections from WT mice after indicated treatments were immunostained for CD31 (red), GFP (green) and DAPI (blue). Quantification of MVD and vessel length is shown on the right, *n* = 3. Data are from three independent experiments and presented as the mean ± s.e.m. Bar = 50 μm. *P < 0.05. ***P < 0.001. Two-tailed unpaired *T*-test

Busulfan, a chemotherapeutic agent used as a conditioning regimen prior to bone marrow transplant, is known to cause azoospermia and infertility^23,24^. Both SSCs and differentiating spermatogonia are killed in mice treated with a single dose of busulfan with the duration of infertility dependent on the extent of SSC depletion^25–27^. At higher busulfan doses, SSCs are ablated preferentially over differentiating spermatogonia and the long delay until spermatogenesis is restored is likely due to destruction of most of the SSC niche limiting factors necessary for self-renewal of the few remaining SSCs. Previous studies have identified GDNF as critical for SSC self-renewal and demonstrated that Sertoli cells and PTM cells are cellular sources of GDNF in the testes and may comprise the SSC niche^10,28–30^. In testis sections, immunostaining for GDNF shows co-localization with TECs and throughout the seminiferous tubules around Sertoli and PTM cells (Supplementary Figure 1a). However, since GDNF is a secreted factor, its localization does not indicate its cellular source. GDNF regulation in the testes is not understood but its expression is dependent in part upon FGF-2^31^. FGF-2 has been suggested to support SSC maintenance by inducing GDNF production by Sertoli cells^32^. To identify GDNF sources in the testes, we quantified *Gdnf* mRNA in Sertoli and TECs cells and measured GDNF levels in media conditioned by TECs or Sertoli cells before and after FGF-2 treatment (Fig. 1a). Since there are significantly fewer TECs than Sertoli cells in the testis^33^, these data indicate that TECs express more GDNF per cell as compared to Sertoli cells, consistent with the notion that TECs are a major GDNF-producing population in the testis^34^. Since GDNF is necessary for the long-term culture of SSCs and Sertoli and PTM cells produce GDNF, we investigated whether Sertoli cells and PTMs could maintain Thy1^+^SSCs in culture. Testicular cells (containing Sertoli and PTM populations) were depleted of CD31^+^ TECs and co-cultured with GFP^+^Thy1.2^+^SSCs. In parallel, GFP^+^Thy1^+^SSCs were also co-cultured with CD31^+^ TECs. After 3 weeks, SSC colonies were absent from testicular cell co-cultures with Sertoli and PTM cells present. In contrast, numerous GFP^+^ Thy1.2^+^ SSC colonies were observed in TEC co-cultures (Fig. 1b).

Studies indicate that Sertoli and Leydig cells are minimally effected by busulfan^26^ (Supplementary Figure 1b). To examine the direct impact of busulfan treatment on TECs in vitro and in vivo, TECs in culture were treated with busulfan and show decreased proliferation and increased apoptosis (Supplementary Figure 1c, d), while testis sections from busulfan-treated mice were immunostained with the EC marker CD31, revealing a significant decrease in microvessel density (Fig. 1c). A decreased TEC population post busulfan treatment is consistent with the global decrease in testis GDNF expression following busulfan exposure (Fig. 1d).

Following exposure to busulfan, restoration of spermatogenesis takes approximately 30–36 weeks, a reflection of the slow proliferation and expansion of the residual SSCs^25,27^. To test whether introduction of healthy TECs can accelerate SCC reconstitution post busulfan, we transplanted syngeneic wild-type primary GFP^+^ TECs into the testes of mice 5 weeks after busulfan administration. To ascertain whether there is a specific requirement for TECs in reconstituting spermatogenesis or whether any organ EC would suffice, we isolated and transplanted either TECs or lung endothelial cells (LuECs) into busulfan-treated testes. The EC identity of the transplanted GFP^+^ TECs and LuECs was confirmed by morphology, immunostaining with EC markers and functionally by tube formation assays (Supplementary Figure 2a, b). By 7 weeks after transplantation (Supplementary Figure 3a), testes harvested from busulfan-treated mice transplanted with TECs were comparable in size to sham injected mice (Fig. 1e). GFP^+^ channels were obvious in whole testes after transplantation with GFP^+^ TECs (Fig. 1e). By contrast, testes in busulfan-treated mice injected with phosphate-buffered saline (PBS) or reconstituted with LuECs remained appreciably smaller (Fig. 1e). Testis sections from busulfan-treated mice, with and without transplanted GFP^+^ TECs, were stained with hematoxylin and eosin and examined for green fluorescent protein (GFP) expression, a germline stem cell marker (PLZF) and a germ cell marker (DDX4) (Fig. 1f). We observed significant restoration of spermatogenesis in testes transplanted specifically with TECs, comparable to that in untreated mice as evidenced by increases in PLZF^+^ and DDX4^+^ cells (Fig. 1f). We also found a significant increase in TEC proliferation, microvessel density and length (Supplementary Figure 3b) and increased proliferation of germ cells in seminiferous tubules (Supplementary Figure 3c). Notably, GFP^+^ cells co-expressed CD31 (Supplementary Figure 3c), confirming the EC identity of the transplanted cells and demonstrating integration of transplanted TECs into the endogenous testis vasculature. Functionality of transplanted GFP^+^CD31^+^ TECs was confirmed by isolectin B4 uptake (Supplementary Figure 4). Remarkably, testes from busulfan-treated mice transplanted with LuECs showed nominal restoration of spermatogenesis (Fig. 1f).

Since our data suggest that TECs promote the restoration of spermatogenesis after busulfan treatment, we examined whether TECs could protect SSCs and spermatogonia from busulfan-mediated cell death by injecting syngeneic wild-type TECs into the testes of mice immediately after busulfan treatment at 3, 6 and 9 days post busulfan injection, and it demonstrated significant protection of spermatogenesis 15 weeks after busulfan treatment. While vehicle-injected mice had no detectable differentiating sperm and very few seminiferous tubules, TEC-injected mice had both differentiating sperm and mature sperm in the lumen of seminiferous tubules as detected by the acrosome-specific marker, lectin-peanut agglutinin^35^ (Supplementary Fig. 5a, b).

### TECs maintain mouse SSCs in culture without exogenous GDNF

**Fig. 2:**
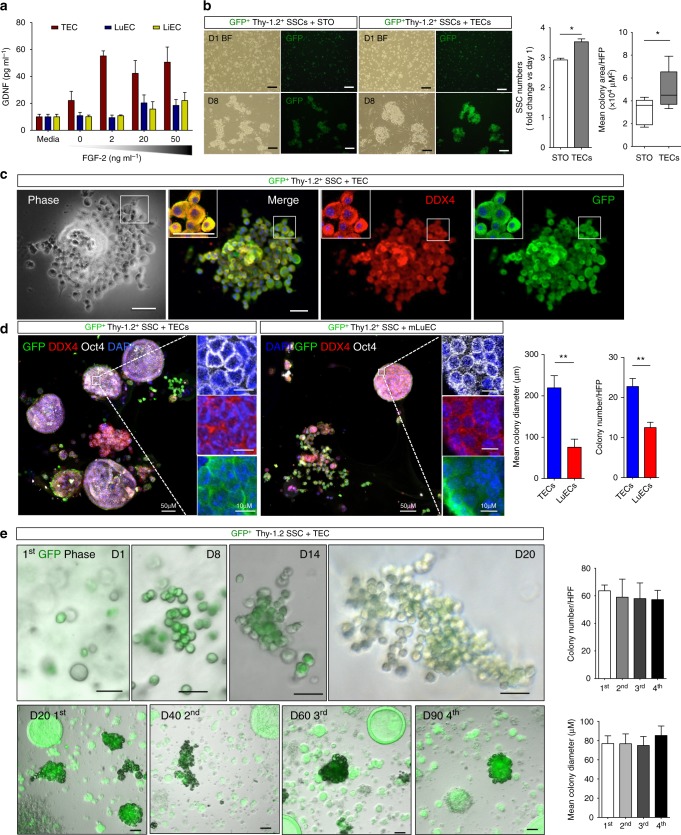
TECs can expand spermatogonial stem cells (SSCs) in culture. **a** Quantification of GDNF by ELISA in conditioned media from TECs, LuECs and LiECs after treatment with the indicated concentration of FGF-2 for 2 days, *n* = 3. Bar = 100 μm. Data are presented as the mean ± s.e.m. **b** Representative bright-field (BF) and GFP images of 2D cultures of GFP^+^ SSCs co-cultured with STO cells or TECs plus exogenous GDNF and FGF-2 on days 1 and 8 after seeding. Quantification of stem cell numbers 8 days after co-culture with STO or TECs and the average colony area are shown on the right, *n* = 3. Data are presented as the mean ± s.e.m. Bar = 100 μm. *P < 0.05. **c** Images of 2D co-cultures of GFP^+^Thy1.2^+^ SSCs with TECs and immunostained with the germ cell marker DDX4. Inset: bar = 10 μm. **d** The 3D spheroid co-cultures of GFP^+^Thy-1.2^+^ SSCs with TECs or LuECs were generated with Matrigel but no exogenous GDNF or FGF-2. GFP^+^ Spheroid co-cultures were immunostained with DDX4 (red), Oct4 (white) and DAPI (blue) at day 15. Quantification of the average diameter and total number of colonies is shown on the right; *n* = 3. All data are presented as the mean ± s.e.m. Bar = 50 μm, *P < 0.05. **P < 0.01. **e** Representative BF images merged with GFP images of serially passaged GFP^+^Thy-1.2^+^ SSCs co-cultured with TECs at the indicated passage number (1st, 2nd, 3rd or 4th) or day (D). Quantification of colony number and mean colony diameter at the indicated passage number is shown on the right. Data shown are representative of six independent experiments. Two-tailed unpaired *T*-test

Since LuECs were unable to restore spermatogenesis in busulfan-treated mice, we compared GDNF production by TECs, LuEC and liver ECs (LiECs). Intriguingly, FGF-2 treatment induced significant levels of GDNF only in TECs, and not in LuECs or LiECs (Fig. 2a), consistent with studies showing ECs exhibit organ-specific gene expression profiles^36,37^. Currently, in vitro cultures of isolated SSCs utilize STO cells or mouse embryonic fibroblasts as feeder cells plus GDNF and several other growth factors^28^. Our data suggest that ECs within the testicular stem cell niche can produce GDNF, a necessary factor to promote SSC proliferation. To confirm the critical supporting role of TECs in the SSC niche, we compared the efficacy of SSC-enriched cultures in vitro maintained either with STO cells or with TECs. After plating SSC-enriched cultures with either TECs or STO cells and adding GDNF and FGF-2 to the serum-free culture media, we monitored the formation of SSC colony number and size. Co-cultures of GFP^+^ SSC-enriched cultures with TECs led to a significant increase in both number and size of colonies compared with STO co-cultures (Fig. 2b). After 5 days, SSC-enriched cultures in the absence of feeder cells did not survive (Supplementary Figure 6a).

To determine whether TECs can support SSC self-renewal and maintenance in the absence of additional GDNF, we assayed three-dimensional (3D) colony-forming capacity of Thy-1.2^+^ SSC-enriched-TEC co-cultures since efficient 3D colony formation is a hallmark of stem cells. GFP^+^Thy-1.2^+^ cells were plated either with Matrigel alone or together with primary mouse TECs in the absence of exogenous GDNF (Supplementary Figure 6b). GFP^+^Thy-1.2^+^ cells co-cultured with TECs and Matrigel formed colonies with typical SSC morphology after 2 weeks as observed by phase contrast microscopy, while SSC-enriched cultures plated in the absence of TECs or GDNF died within 7 days (Fig. 2c and Supplementary Figure 6b, c). The stem cell identity of these colonies was confirmed by expression of the germ stem cell marker DDX4 (Fig. 2c). Next, we co-cultured an excess of GFP^+^Thy-1.2^+^ cells to TECs and Matrigel with these cultures demonstrating significant numbers of large, spheroid colonies as early as 2 weeks after plating. Colonies were immunostained for the expression of the germ stem cell markers DDX4 and Oct4 (Fig. 2d and Supplementary Figure 6d). Of note, spheroid colonies generated by co-cultures of GFP^+^Thy-1.2^+^ SSC-enriched cells with LuECs were significantly smaller in diameter and fewer in number than colonies formed with TECs (Fig. 2d). The 3D colonies with an SSC-enriched population and TECs were maintained for greater than 3 months in culture in the absence of exogenous GDNF, over which time the colonies continue to proliferate, indicating that TECs may be sufficient to maintain SSCs in vitro. Moreover, these putative GFP^+^ SSC/TEC colonies were dissociated and serially passaged multiple times in the presence of TECs without measurable decline in colony-forming efficiency (Fig. 2e).

### Transplanting SSCs co-cultured with TECs restores fertility

**Fig. 3:**
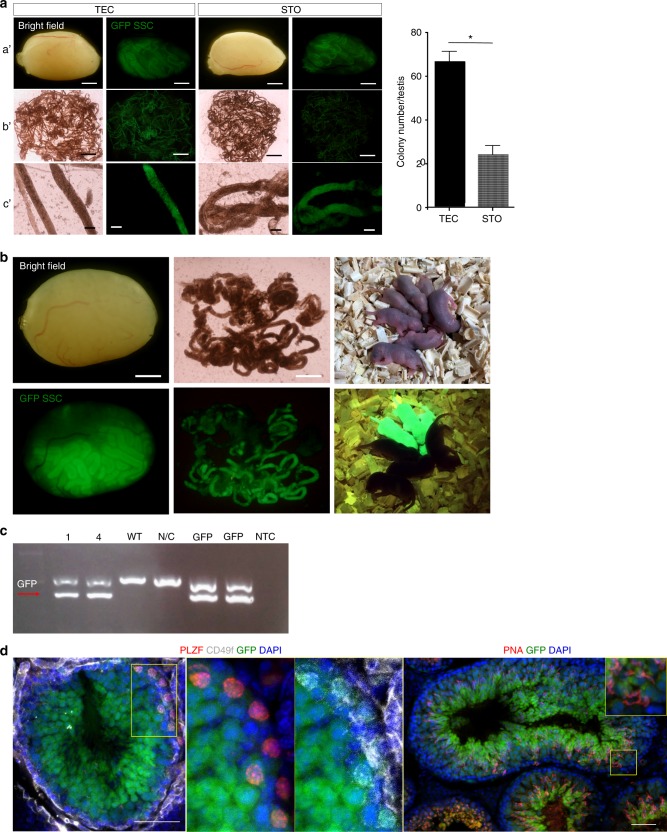
SSCs cultured long term with TECs restore spermatogenesis and fertility in mice. **a** Images of testes from busulfan-treated mice 12 weeks after transplantation with GFP^+^ SSCs co-cultured long term with either TECs or STO feeder cells. Quantification of GFP^+^ SSC colonies is shown on right. Data shown are out of three independent experiments. a’: Bar = 2 mm, b’: bar = 4 mm, c’: bar = 200 μm. *P < 0.05. **b** Images of testes and GFP^+^ pups 16 weeks after transplantation of GFP^+^ SSCs co-cultured long term with TECs into infertile W/W^v^ mice. Bar = 2 mm. **c** PCR of *gfp* expression in GFP^+^ pups (1, 4) non-GFP^+^ pups (WT), negative control (N/C), positive controls (GFP, GFP) and a no DNA template control (NTC). **d** Images of testis sections immunostained with the undifferentiated spermatogonia markers PLZF and Cd49f as well as the spermatid marker PNA. Testes were from mice transplanted with GFP^+^ SSCs after long-term co-culture with TECs. Data shown are out of six independent experiments. Two-tailed unpaired *T*-test

Since murine SSCs can expand and self-renew on STO feeder cells, we compared the function of murine SSCs expanded on TECs versus STO cells. First, we examined colony formation by cultured SSCs in vivo, by transplanting equal number of GFP^+^ SSCs co-cultured with TECs or STO feeder cells into testes of busulfan-treated mice (Supplementary Figure 6e). At 12 weeks after transplantation, recipient mice testes were analyzed for colony formation of GFP^+^ SSCs co-cultured with either TECs or STO cells (Fig. 3a). We found that testis transplanted with SSCs co-cultured with TECs showed significantly more colonies than testis transplanted with SSCs co-cultured with STO (Fig. 3a).

The ultimate confirmation of SSC function is the ability of infertile mice to give birth to live offspring after SSC transplantation. GFP^+^ SSCs co-cultured long term with TECs were transplanted into W/W^v^ mice which lack germ cells and are infertile^38,39^. At 16 weeks after transplantation, GFP^+^ SSC colonization of the testes was observed as well as the birth of GFP^+^ pups (Fig. 3b) with *Gfp* expression in pups confirmed by PCR (Fig. 3c). Testis sections from mice transplanted with SSCs after long-term culture were immunostained with the undifferentiated spermatogonia markers PLZF and CD49f as well as the spermatid marker peanut agglutinin (PNA), further demonstrating functional spermatogenesis of transplanted GFP^+^ SSCs (Fig. 3d).

### GDNF is regulated by an FGF-2–FGFR1–CaN–NFAT signaling axis

The mechanism of GDNF regulation in the testes is not well understood but its expression is known to be, at least in part, dependent upon FGF-2^31^. FGF-2 has been suggested to promote SSC maintenance by inducing GDNF production by cells in the testes. Quantification of GDNF levels in media conditioned by primary TECs after FGF-2 treatment indicate that TECs produce GDNF. Our data show FGF-2 treatment induced significant levels of GDNF only in TECs, and not in LuECs or LiECs, consistent with studies showing ECs exhibit organ-specific gene expression profiles^36,37^.

**Fig. 4:**
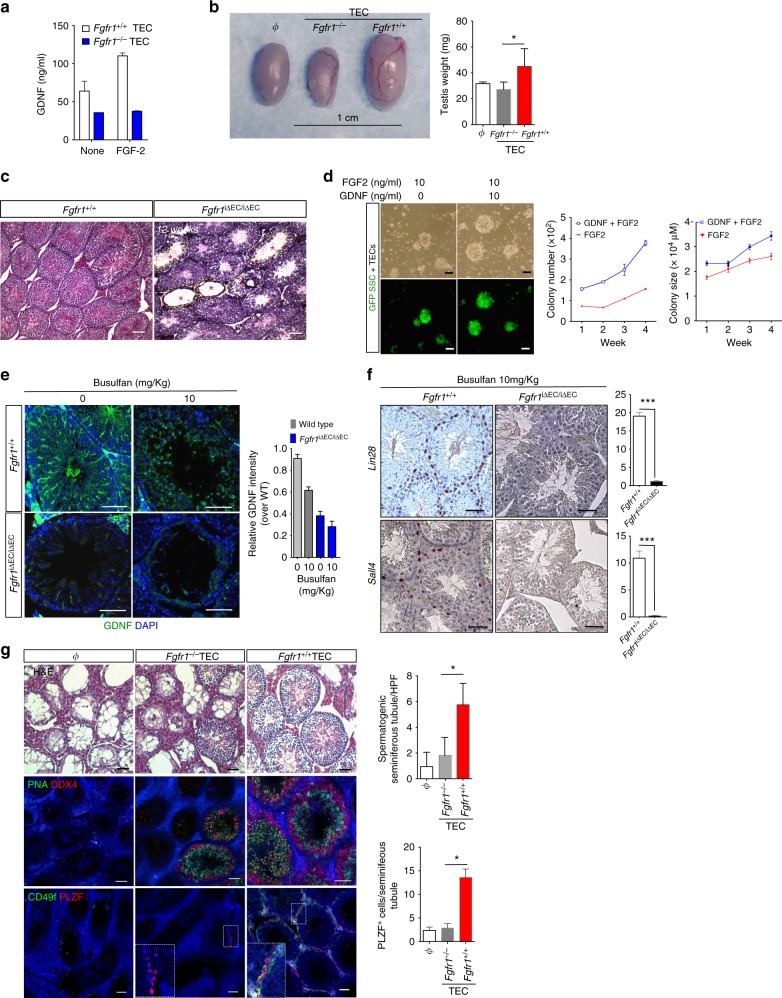
FGF-2 activates GDNF expression in TECs via FGFR1. **a** Quantification of GDNF levels by ELISA in conditioned media from TECs isolated from *Fgfr1*^+/+^ or *Fgfr1*^−/−^ mice. Bar = 1 cm. **b** Images of testes from busulfan-treated (45 mg kg^−1^) WT mice 12 weeks post transplantation with PBS (Φ), *Fgfr1*^+/+^ TECs or *Fgfr1*^−/−^ TECs. Testis weights are indicated on the right (*n* = 4). **c** H&E images of testis sections from control (*Fgfr1*^+/+^) or endothelial-specific deletion of *Fgfr1* (*Fgfr1*^iΔEC/iΔEC^) mice at 12 weeks after tamoxifen treatment to induce fgfr1 deletion from ECs. Red stars indicate empty seminiferous tubule. Bar = 100 μm. **d** Representative bright-field and GFP images of GFP^+^ SSCs co-cultured with TECs with the addition of FGF ± GDNF at the indicated concentrations after 4 weeks. SSC colony number and size over time are shown on right. **e** Immunofluorescence images of testis sections from *Fgfr1*^+/+^ or *Fgfr1*^iΔEC/iΔEC^ mice 5 weeks after busulfan treatment at the indicated concentrations. Sections were immunostained for GDNF with relative GDNF intensity quantified on the right (*n* = 3). **f** Representative images of testis sections immunostained for the SSC markers Lin28 and Sall4 from *Fgfr1*^+/+^ (*n* = 3) or *Fgfr1*^*iΔEC/iΔEC*^ (*n* = 4) mice 5 weeks after busulfan treatment (10 mg kg^−1^). Sall4^+^ or Lin28^+^ cells are quantified on the right. **g** Representative images of testis sections from WT mice (*n* = 3–4) treated with busulfan and transplanted with PBS (Φ), *Fgfr1*^−/−^ TEC or *Fgfr1*^+/+^ TEC. Sections were stained with H&E and immunostained for Lectin-PNA, DDX4, CD49f and PLZF. Quantification of spermatogenic seminiferous tubules and PLZF^+^ cells are on the right. All data are presented as the mean ± s.e.m. Bar = 50 μm. *P < 0.05, ***P < 0.001. Data shown are representative of two independent experiments. Two-tailed unpaired *T*-test

FGF-2 activation of ECs occurs primarily through binding to FGFR1^40^. To determine the requirement for FGFR1 expression on TECs for GDNF production, we isolated TECs from our mouse model of inducible *Fgfr1* deletion in ECs, referred to as *Fgfr1*^iΔEC/iΔEC^ mice. *Fgfr1*^iΔEC/iΔEC^ TECs were treated with FGF-2 and subsequently GDNF levels were measured in the media. *Fgfr1*^*−*^^/−^ TECs showed no increase in GDNF expression after FGF-2 treatment as compared to *Fgfr1*^*+/+*^ wild-type TECs (Fig. 4a). To examine the effect of *Fgfr1* deletion in TECs^8^ on spermatogenesis in vivo, wild-type male mice were treated with busulfan, and 5 weeks later transplanted with *Fgfr1*^*−/−*^ TECs, *Fgfr1*^*+/+*^ TECs or PBS alone. Testes harvested from mice 12 weeks after transplantation with *Fgfr1*^*−/−*^ TECs were comparable in size and weight to testes that were injected with PBS alone and significantly smaller in size and weight than testes transplanted with *Fgfr1*^*+/+*^ TECs (Fig. 4b). Testis sections were stained with hematoxylin and eosin (H&E) and demonstrate a significant number of seminiferous tubules lacking developing sperm (Fig. 4c). We also observed a significant decrease in PLZF^+^ cells and ECs as well as a reduction in proliferating germ cells in testes from *Fgfr1*^iΔEC/iΔEC^ mice (Supplementary Figure 7a, b). Our data implicate FGF-2 binding to FGFR1 is a key regulator of GDNF expression in TECs, and thus we co-cultured Thy1.2^+^GFP^+^ cells with TECs and added FGF-2 alone or with GDNF (Fig. 4d). While colony formation was robust as quantified by both number and size of colonies in the co-cultures supplemented with FGF-2 and GDNF, the addition of FGF-2 alone to the growth media was sufficient for SSC maintenance and expansion (Fig. 4d) presumably due to the presence of TEC-derived GDNF.

Collectively, our data suggest that GDNF production should be impaired in mice with an endothelial-specific deletion of *Fgfr1* affecting SSC self-renewal after busulfan-mediated injury. Wild-type and *Fgfr1*^iΔEC/iΔEC^ mice were treated with busulfan and 4 weeks later testis sections were immunostained for GDNF showing significantly decreased expression in the testes of *Fgfr1*^iΔEC/iΔEC^ mice even after low-dose busulfan treatment (Fig. 4e). Since expansion of residual SSCs after low-dose busulfan treatment requires GDNF, 4 weeks after busulfan treatment, we examined the presence of SSCs in testis sections from *Fgfr1*^iΔEC/iΔEC^ mice by immunostaining for the SSC markers, Lin28 and Sall4. In contrast to wild-type mice, there was an almost complete absence of both Lin28^+^ and Sall4^*+*^ cells in testis sections from *Fgfr1*^iΔEC/iΔEC^ mice (Fig. 4f and Supplementary Figure 7c). If FGFR1 expression is necessary for GDNF production, transplanting *Fgfr1*^*−/−*^ TECs should not restore spermatogenesis in busulfan-treated mice. Indeed, H&E sections of testes harvested from busulfan-treated mice after transplantation of *Fgfr1-*null TECs showed minimal spermatogenesis and few DDX4- or PLZF-positive cells as compared to mice transplanted with *Fgfr1* wild-type TECs (Fig. 4g). Immunostaining with markers of differentiating sperm PNA and CD49f further confirmed the inability of *Fgfr1*^*−/−*^ TECs to re-establish robust spermatogenesis in busulfan-treated mice (Fig. 4g).

### Spermatogenesis defects in DS mice

**Fig. 5:**
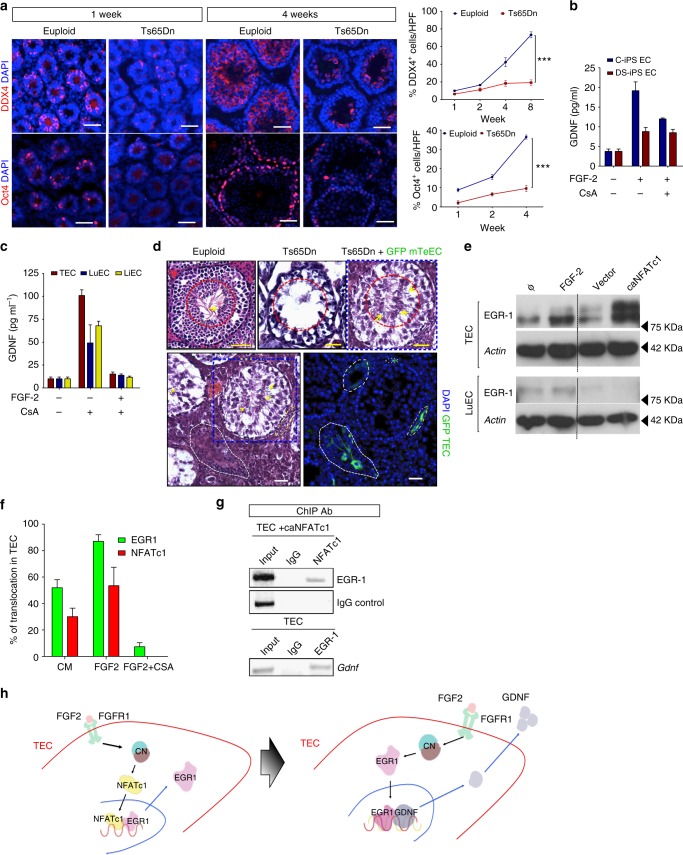
GDNF expression in TECs is regulated by CaN–NFAT signaling. **a** Immunofluorescence images of testis sections immunostained with the stem cell markers DDX4 and Oct4 and DAPI to stain DNA. Testes were harvested from 1-week-old or 4-week-old Euploid or Ts65Dn mice. Quantification of DDX4^+^ and Oct4^+^ cells relative to DAPI per high-powered field (HPF) is shown on the right, ***P < 0.0001. Bar = 50 μm. Two-way ANOVA test. **b** Quantification of GDNF levels by ELISA in conditioned media from control iPS-derived ECs (C-iPS EC) and Down syndrome iPS-derived ECs (DS-iPS-ECs) after 2 ng ml^−1^ FGF-2 treatment in the presence or absence of cyclosporin A (CsA). Values are mean ± s.e.m, *n* = 4. **c** Quantification of GDNF levels by ELISA in conditioned media from murine TECs, LuEC and LiEC after 2 ng ml^−1^ FGF-2 treatment in the presence or absence of CsA. Values are mean ± s.e.m., *n* = 4. **d** H&E-stained cross-sections from testes of age-matched Euploid, Ts65Dn or Ts65Dn mice 5 weeks after implantation with GFP^+^ TECs isolated from syngeneic wild-type mice (Ts65Dn+GFP^+^ TECs). Red dotted line encircles mature sperm (yellow arrow) in the lumen of the seminiferous tubules. Immunofluorescence images with GFP and DAPI to stain DNA of testis sections from Ts65Dn+GFP^+^TECs mice. **e** Western blot analysis of EGR-1 protein expression in TECs and LuECs untreated or after FGF-2 treatment, expression of constitutively active NFATc1 (caNFATc1) or an empty vector control. Actin was probed as a loading control. **f** Quantification of nuclear translocation of EGR1 and NFATc1 in TECs after treatment with conditioned media (CM), 2 ng ml^−1^ FGF-2±CsA. Values are mean ± s.e.m. **f** Chromatin immunoprecipitations (ChIP) of TECs expressing caNFATc1 with anti-NFATc1 mAb or of TECs with anti-EGR-1 antibody. NFATc1 or EGR-1 was immunoprecipitated and DNA probed by PCR for NFATc1 consensus sites on the *Egr1* promoter or EGR1 consensus sites on the *Gdnf* promoter. IgG pulldown was used as a control. **h** Schematic of GDNF regulation via FGF-2-CaN-NFATc1-EGR-1 in TECs

Our data indicate that GDNF production by TECs is induced by FGF-2 binding to FGFR1. The most common signaling pathway downstream of FGF-2 is the MAP kinase pathway; however, other pathways have been linked to FGF-2–FGFR1 signaling including the calcium activated CaN–NFAT axis^40^. We and others have previously shown that endothelial activation is impaired in DS due in part to chromosome 21-encoded inhibitors of CaN–NFAT signaling^20^. Other studies show that males with DS have significantly reduced sperm counts and are either subfertile or infertile^21,22^. The Ts65Dn DS mouse model with segmental human trisomy has many features of DS including male infertility^41,42^. An almost complete lack of developing sperm throughout the seminiferous tubules and in the lumens along with an overall decrease in tubule size is observed by 8 weeks in these mice (Supplementary Figure 8a). To identify the SSC population, we immunostained testis sections for the germ cell markers DDX4, which labels SSCs and differentiating sperm, as well as OCT4 and PLZF, primordial germ cell markers^43^, and the proliferation marker Ki67. The Ts65Dn testes exhibited decreased DDX4-, PZLF- and OCT4-positive cells over time as compared to euploid littermates (Fig. 5a and Supplementary Figure 8b). Examination of Sox9, a marker of Sertoli cells, revealed moderate decreases in this population in Ts65Dn testes versus euploid controls (Supplementary Figure 8c).

To further investigate the role of TECs and associated spermatogenic defects of DS with a more tractable system, we used human induced pluripotent stem cells (iPS) from Trisomy 21 (DS) and control human subjects to generate ECs. Trisomy 21 was confirmed by karyotype (Supplementary Figure 9a), while EC identity was validated by immunostaining with EC markers VEGFR2, CD31, VE-cadherin and von Willebrand factor and functionally by capillary tube formation and acetylated low-density lipoprotein (LDL) uptake (Supplementary Figure 9b). ECs derived from DS-iPS-ECs failed to organize into tube-like structures on Matrigel and exhibited defective proliferation and migration as compared to control (C) iPS-derived ECs in response to VEGF (Supplementary Figure 9b-d and Supplementary Movie a,b). Analysis of the secretome of C- and DS-iPS-derived ECs identified a dramatic decrease in GDNF expression (Supplementary Figure 9e). Previously, we have shown that defects in ECs derived from the Ts65Dn mouse model of DS were due to attenuation of CaN–NFAT signaling^20^. To examine the effect of FGF-2 on GDNF production by both C- and DS-iPS-ECs, we treated both groups with FGF-2 and measured GDNF production in conditioned media. In C-iPS EC, FGF-2 stimulated robust GDNF production that was significantly abrogated in the presence of the specific CaN inhibitor cyclosporin A (CsA). However, in DS-iPS EC, the relatively modest induction of GDNF by FGF-2 was unaffected by CsA (Fig. 5b). FGF-2 treatment also stimulated significant GDNF production in a CaN-dependent fashion in primary ECs specifically from mouse TECs and to a much lesser extent in mouse LuECs and LiECs (Fig. 5c). To further validate the importance of TEC-derived GDNF in the SSC niche, we transplanted wild-type GFP^+^ TECs into the testes of Ts65Dn DS mice and showed restoration of spermatogenesis (Fig. 5d).

In astrocytes, FGF-2 regulates GDNF production by inducing the expression of early growth response protein 1 (EGR-1), a transcription factor that binds the *Gdnf* promoter^44^. *Egr-1* and *Nfat* family members synergize to activate gene expression in numerous tissues^45,46^. We postulated that FGF-2 binding to FGFR1 activates CaN with subsequent cooperation between NFAT and EGR-1 to promote GDNF expression by TECs. Western blot analysis confirmed increased EGR-1 expression specifically in TEC and not LuEC after FGF-2 treatment or upon expression of a constitutively active nuclear *Nfatc1* construct (*caNfatc1*) (Fig. 5e) that also increases GDNF production by TECs (Supplementary Figure 10a). Similarly, in DS-iPS EC with attenuated calcineurin signaling^20^, there was minimal EGR-1 expression after FGF treatment but expression of a caNFATc1 construct did upregulate EGR-1 (Supplementary Figure 10b). Previous studies have shown that *Nfatc1* induces *Egr-1* in a CaN-dependent manner^44,45^; thus, to determine whether EGR-1 upregulation was dependent on NFATc1 activation, the subcellular localization of NFATc1 and EGR-1 was assessed in TECs after FGF-2 treatment with and without CsA (Fig. 5f and Supplementary Figure 10c). While the nuclear localization of both NFATc1 and EGR1 increased after the addition of FGF-2, CsA inhibited FGF-2-induced EGR-1 nuclear localization, indicating CaN dependence (Fig. 5f and Supplementary Figure 10c). Additionally, an NFATc1 chromatin immunoprecipitation (ChIP) shows that *caNfatc1* binds to the *Egr1* promoter but not the *Gdnf* promoter in TECs, while an EGR-1 ChIP indicates direct EGR-1 binding to the *Gdnf* promoter in TECs (Fig. 5g and Supplementary Figure 10d, e). Collectively, our data support a model whereby FGF-2 binding to FGFR1 on TECs activates a CaN–NFAT–EGR1–GDNF pathway regulating GDNF production by TECs (Fig. 5h).

### Human SSCs are maintained in long-term culture

**Fig. 6:**
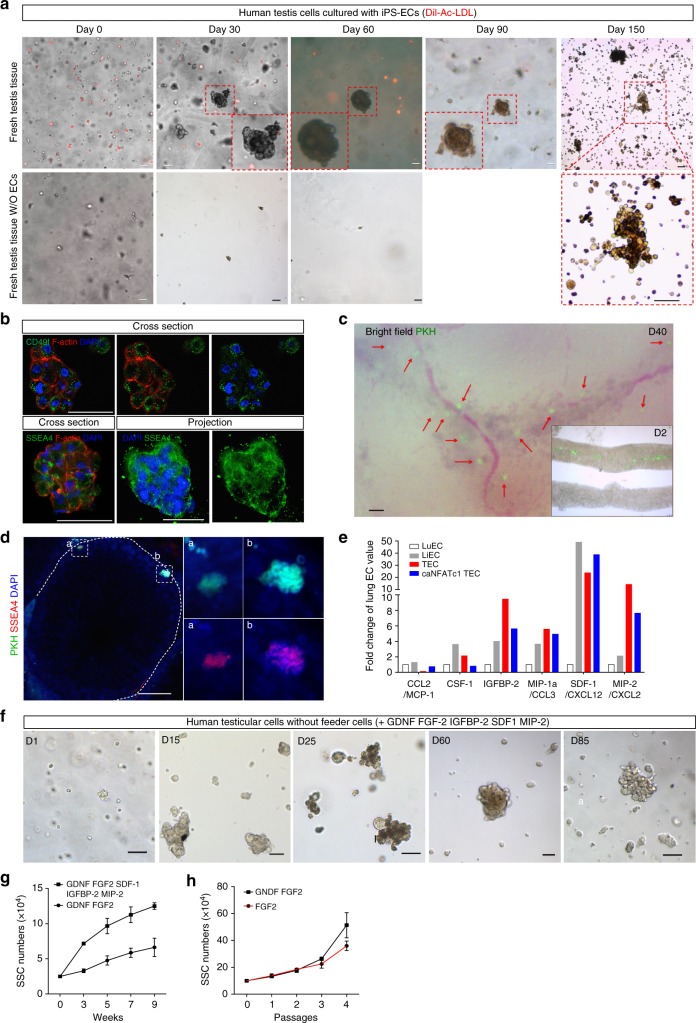
Human SSCs can be maintained and expanded in vitro with human ECs. **a** Representative brigh-tfield images of human testicular cells from fresh and frozen testis tissue cultured with or without human ECs after Dil-Ac-LDL (red) uptake on the indicated days. Bar = 100 μm. **b** Characterization of SSC colonies generated in human testicular cell and endothelial cell co-cultures. Colonies were immunostained with the human SSC markers, CD49f and SSEA4, Bar = 50 μm. **c** Bright-field and GFP-merged images at day 2 (inset) and day 40 after transplantation of PKH-labeled human SSC colonies (green) expanded in co-cultures with ECs, into busulfan-treated immunodeficient Nude mice. Red arrows indicate PKH-labeled SSC colonies in the seminiferous tubules. Transplanted PKH-labeled human SSC colonies (green) were visible in the cavity of seminiferous tubules at day 2 after microinjection. **d** Representative immunofluorescence images of cross-sections of a seminiferous tubule from Nude mice 40 days after transplantation with PKH-labeled human SSC colonies (green). Sections were immunostained with SSEA4 (red). Bar = 50 μm. **e** Relative expression of secreted factors by antibody arrays in media conditioned by LuEC, LiEC, TEC and TECs expressing constitutively active NFATc1 (caNFATc1) during Matrigel capillary tube formation. **f** Representative bright-field images of human testicular cells showing SSC colonies in feeder-free cultures in media containing SDF-1, MIP-2, IGFBP-2, GDNF and FGF-2 at the indicated days, bar = 50 μm. **g** Quantification of SSC numbers over time in feeder-free cultures with the indicated growth factors. **h** Quantification of SSC numbers in co-culture with human TECs and addition of either FGF-2 alone or FGF-2 and GDNF at the indicated passages. Values are mean ± s.e.m, *n* = 3. Data shown are representative of three independent experiments

Numerous studies have cultured murine SSCs for long term and transplanted them into busulfan-treated infertile male mice^11^. However, studies demonstrating long-term culture of human SSCs have been limited and not easily reproduced^47,48^. To determine whether human SSCs expanded on ECs retain their stem-like properties, we obtained testicular biopsies from prepubertal boys diagnosed with cancer prior to the onset of treatment. Due to the very small sample size of these biopsies, we plated the entire sample of testicular cells onto a monolayer of human ECs to minimize any loss of SSCs through selection. Because we were unable to isolate TECs from these minute testicular biopsies, we utilized human iPS-derived ECs labeled by Dil-Ac-LDL uptake. Using both fresh and previously frozen testicular biopsies, we examined putative SSC colony formation in vitro over time cultured with either iPS-ECs (Fig. 6a) or without ECs. By day 30, both fresh and frozen testicular cells co-cultured with iPS-ECs displayed classic SSC-like colonies throughout the culture, while freshly isolated testicular biopsies plated in the absence of ECs but supplemented with GDNF and FGF-2 died after 2 weeks. Both freshly isolated and previously frozen SSC-like cells with ECs continued to expand over time with numerous SSC-like colonies throughout the cultures observed at day 150 (Fig. 6a and Supplementary Figure 11a). To validate the presence of SSCs in colonies from our testicular cell/EC co-cultures, we examined expression of human SSC markers. Sections were immunostained for SSEA4 and CD49f/ITGA6, markers expressed predominantly on SSCs^49–52^. Immunofluorescence analysis shows that colonies from human testicular cell/EC co-cultures are positive for SSEA4 and CD49f (Fig. 6b), implicating the presence of SSCs in these colonies. To ensure that human SSCs expanded during long-term EC co-cultures retain their stem cell identity, we dissociated SSC-like colonies 150 days after culture in vitro and labeled them with the fluorescent dye PKH67. Our PKH-labeled putative SSCs were transplanted into busulfan-treated Nude mice. The testes were examined after transplantation of our labeled human SSCs (Fig. 6c). At 2 days after transplantation, the PKH67^+^ cells were visible in the seminiferous tubules (Fig. 6c). By 40 days post transplantation, the formation of PKH67^+^ colonies in the seminiferous tubules were evident (Fig. 6c). Testes were isolated, sectioned and immunostained positively for SSEA4, a SSC marker, indicating the presence of SSCs in PKH67^+^ colonies (Fig. 6d).

Our data suggest that TECs, but not other organ endothelium, can support SSC self-renewal. Thus, we compared the secretome of TECs to that of LuECs and LiECs during tube formation assays to identify unique factors produced during TEC activation that may be critical for SSC maintenance. Besides GDNF, we identified three other factors that were specifically upregulated in TECs and also upregulated in TECs expressing caNFATc1 implicating their CaN dependence (Fig. 6e). These factors, IGFBP-2, SDF-1 and MIP-2, have been implicated in stem cell biology and hence were added along with GDNF and FGF-2 to human testicular cells or mouse testicular cells (Supplementary Figure 11b) in vitro in the absence of feeder cells. By day 15, SSC colonies with typical morphology were observed in our feeder-free cultures. By day 25, robust SSC colony formation was observed that were maintained even after 85 days in culture (Fig. 6f). Quantification of SSC numbers in feeder-free cultures show that addition of GDNF, FGF-2, IGFB2, SDF-1 and MIP-2 supported significant increases in SSC numbers over 9 weeks in comparison to cultures with only GDNF and FGF-2 (Fig. 6g). The ability to culture and expand SSCs under feeder-free conditions has important clinical significance by allowing the expansion of SSCs from testicular biopsies obtained from prepubertal boys prior to cancer treatment for restoration of fertility after a cancer-free diagnosis. To confirm that human SSCs continue to grow on human TECs, we plated entire testicular biopsy samples onto human TECs and show continued expansion of SSCs cultured with human TECs over multiple passages with no difference in growth rates between addition of exogenous FGF-2 alone or FGF-2 with GDNF (Fig. 6h). Collectively, our data support our hypothesis that human TECs are a critical source of GDNF and other factors that are necessary and sufficient to support the self-renewal and expansion of human SSCs in vitro. This provides a feasible approach to expand the minute number of human SSCs present in testicular biopsies obtained from prepubertal boys for later transplantation to restore spermatogenesis and fertility.

## Discussion

It has long been established that stem cells, a self-renewing population of cells in most organs, are maintained in specialized tissue niches that require heterotypic supporting cells to provide factors necessary for their maintenance^1,2^. However, for most organs, including the testis, the accessory cells required for stem cell self-renewal have not yet been conclusively identified. Collectively, our data implicate the testicular endothelium as a key population in the SSC niche producing GDNF and other factors necessary for SSC self-renewal. Here we show that TECs can maintain and expand putative SSCs in long-term culture, restoring spermatogenesis in mice after chemotherapy-induced infertility. SSCs cultured long term with TECs were functional as demonstrated by the birth of live pups after transplanted GFP^+^ SSCs. Further, we identified five growth factors produced specifically by TECs, but not other organ endothelium that are sufficient to maintain SSC-like colonies in feeder-free cultures. Our studies also provide insight into the mechanisms regulating GDNF expression in TECs by demonstrating FGFR1–CaN–NFAT signaling as the key pathway.

For many years, it was assumed that endothelium throughout the body was functionally redundant. However, more recently studies have shown that ECs in different organ environments have distinct properties and roles with organ-specific functions regulated in part by unique secretomes. For example, liver endothelium has been shown to underlie liver regeneration by its production of hepatocyte growth factor, while lung endothelium is required for lung regeneration after injury due to its expression of MMP14^8,9^. It is becoming increasingly evident that ECs from different organs are not interchangeable. Our data clearly indicate specialized roles for TECs in the germ cell niche that cannot be replaced by ECs from other tissues. Further confirmation of the significance of TECs in the SSC niche was observed by the restoration of spermatogenesis observed in DS mice after transplantation of wild-type TECs into the testis of Ts65Dn DS mice. Our data indicate defects in TEC activation in DS due to attenuation of the CaN–NFAT pathway by chromosome 21-encoded inhibitors of this pathway preventing transactivation of the NFAT-dependent target, GDNF.

GDNF has been identified as the single most important factor in SSC self-renewal as its loss leads to impaired spermatogenesis and its overexpression to expansion of undifferentiated spermatogonia in transgenic mouse models^10^. Other cells in the testis such as Sertoli cells and PTM cells are also thought to produce GDNF^53,54^. However, our data show that the restoration of murine spermatogenesis requires 6-month post-busulfan treatment due to the slow expansion of the few surviving SSCs^27^, while transplantation of TECs into the testis after busulfan-induced SSC loss restores spermatogenesis within weeks. Further, injection of wild-type TECs into mice immediately after busulfan treatment is sufficient to protect SSC destruction indicating a pro-survival function for TECs. While it is likely that the contribution of GDNF from Sertoli and PTM cells are also necessary for SSC maintenance, the level of GDNF produced from these two populations in the testes may not be sufficient. Since FGF-2 is also necessary for the maintenance of SSCs, we propose that FGF-2 activates TECs to produce GDNF and other factors for SSC self-renewal and/or maintenance. A critical threshold of GDNF is necessary for SSC maintenance but the source of GDNF may be less critical with TECs, Sertoli cells and PTMs all required to produce sufficient GDNF for SSC self-renewal.

Although murine SSCs can be expanded in culture, the ability to reproducibly culture human SSCs in the long term has not yet been achieved^12,47^. The lack of fertility preservation options for prepubertal boys diagnosed with cancer has been attributed to the inability to reproducibly expand the minute SSC population in testicular biopsies obtained from these patients prior to the onset of cancer treatment. Here our data indicate that human SSCs can be maintained in co-culture with human TECs in the long term. Functional confirmation of long-term cultured human SSCs was observed by migration to the basement membrane of the seminiferous tubules of Nude mice after transplantation. We show that TECs may be sufficient for the self-renewal and expansion of SSCs; however, the clinical application of stem cells is often hindered by a requirement for feeder cells. Since supplementation of GDNF alone is not sufficient for SSC survival when cultured on STO cells, we screened the secretome of activated TECs in comparison to liver and lung ECs. We identified IGFBP-2, SDF-1 and MIP-2 as specifically produced by TECs and previously implicated in stem cell biology^55,56^. Our work shows that the addition of IGFBP-2, SDF-1 and MIP-2 along with GDNF and FGF-2 is sufficient for the expansion and long-term culture of both murine and human SSCs in the absence of feeder cells.

Collectively, our data provide evidence for TECs as a key population in the germ cell niche providing GDNF, IGFBP-2, SDF-1 and MIP-2 for the maintenance of SSCs. Delineating the contribution of the CaN–NFAT pathway in regulating GDNF production in TECs presents therapeutic targets for male infertility. Of great clinical significance is the identification of five growth factors that permit feeder-free expansion of SSCs in vitro removing the risk of transmitting feeder cell-derived viruses to SSCs. These data will allow us to expand human SSCs obtained from testicular biopsies of prepubertal boys diagnosed with cancer prior to the onset of cancer treatment with the possibility of preserving fertility in this patient population by eventually reintroducing SSCs upon treatment completion and a cancer-free diagnosis.

## Methods

### Cell viability assay

1 × 10^3^ TECs or Sertoli cells (Lonza) were plated in 96-well plates and incubated at 37 °C for 24 h. The 96-well plates assays were coated with 0.1% gelatin prior to seeding. Busulfan (Sigma) was diluted in dimethyl sulfoxide (DMSO) used at the indicated concentrations and incubated for 96 h. Media were removed and 100 μl of MTT working solution (ScienCell^TM^) was then added to each well followed by incubation at 37 °C for 2 h. The MTT working solution was removed and 50 μl of DMSO was added to each well. Samples were read at 550 nm wavelength. Each condition was performed in triplicate with *n* = 3 experiments. Statistics were analyzed with GraphPad Prism.

### TEC proliferation and apoptosis assays

5 × 10^3^ testicular ECs per well were plated on 8-chamber slide (LabTeK) coated with 0.1% gelatin. Busulfan (800 μM) or DMSO were added to each well 24 h after seeding and cultured for 72 or 96 h. Then, 10 μM BrdU (BD Pharmingen^TM^) was added to media 72 h after busulfan treatment and then incubated for 2 h. Samples were stained with anti-BrdU (1:50; Invitrogen), anti-γ-H2AX (1:400; Millipore) and anti-cleaved caspase 3 (1: 400; Cell Signaling). BrdU^+^ cells in 10 random high-powered fields were counted and statistics were analyzed using GraphPad Prism. Each condition was performed in triplicate.

### Human testicular cells

Human testicular cells from prepubertal boys were obtained through open testicular biopsies performed by an urologist during a procedure when the patient is under general anesthesia for another purpose, i.e., central line placement, bone marrow aspirates/biopsies. This procedure occurs before any cancer therapy is initiated. A small incision is made in the superior pole of the testis and an approximately 80 mm^3^ portion of the extruded seminiferous tubules is excised (about 2 mm × 4 mm × 10 mm). The size varies depending on the size of the patient. Consent was obtained prior to obtaining testicular biopsies. Given the young age of these patients, their parents signed the informed consent form and assent was obtained from the patients over the age of 12 years. All procedures were approved by the institutional review board at the Children’s Hospital of Philadelphia.

### Isolation of TECs

To isolated TECs, testes from 3–4-week-old mice were harvested and minced as fine as possible. Testes tissues were digested in Hanks' balanced salt solution (HBSS) supplemented with 10 mg ml^−1^ of type collagenase (Worthington) and 20 µg ml^−1^ of DNase I (Sigma) for 35 min at 37 ^o^C with shaking over 250 rpm. Digested tissues were collected by spinning down 1500 rpm for 5 min at 4 ^o^C and tissue pellets were resuspended in 0.25% tyrpsin added with 7 mg ml^−1^ of DNase I (Worthington), then incubated at 37 ^o^C for 5 min. Separated cells were then strained sequentially through a 100 µm and a 40 µm strainer and trypsin activity was quenched with equal volume of fetal bovine serum (FBS). Isolated cells were washed once with 10 ml of HBSS and spindown at 1000 rpm for 10 min at 4 ^o^C. The cells were resuspended in 100–200 µl of MACS buffer and mixed with 10–20 µl of CD31 microbead (Miltenyi Biotec: Cat: 130-097-418) and 10–20 µl of mouse Fc receptor blocker and then incubation for 15 min at 4 ^o^C. Antibody binding cells were isolated by MACS cell separation following the manufacturer’s instruction. Purified cells were confirmed by immunostaining with CD31 (1:50, BD; Cat: 553370), VEGFR2 (1:100; Cell signaling; Cat: 55B11) and VE-cadherin (1:100, Santa Cruz; Cat: SC-9989), which were specifically expressed on ECs and Dil-Ac-LDL uptake assay (Alfa Aescar).

### SSC cultures with TECs or TEC-negative testicular cells

C57BL/6J mice were obtained from Harlan Laboratories (Indianapolis, IN, USA). All mice were used at 2–3 weeks of age. For the isolation of TECs and TEC-negative testicular cells, magnetic-activated cell sorting (MACS) with anti-CD31 and anti-Thy-1 microbeads was conducted (Miltenyi Biotech, Auburn, CA, USA). Briefly, fresh testes were placed in Dulbecco’s phosphate-buffered saline (DPBS; Invitrogen, Grand Island, NY, USA) and decapsulated. Seminiferous tubules were then incubated in a 4:1 solution of collagenase type II (Worthington) 10 mg ml^−1^ and 0.5 mg ml^−1^ DNAse I (Worthington) in DPBS at 37 °C for 30 min. Cells were centrifuged at 1500 rpm for 5 min at 4 °C and then incubated in a 4:1 solution of 0.25% trypsin-EDTA (Invitrogen) and 7 mg ml^−1^ DNAse I (Roche, Basel, Switzerland) in DPBS at 37 °C for 5 min. Enzyme digestion was inactivated by the addition of FBS (Biotechnics research, INC. USA) equivalent to 10% of the initial reaction volume. After digestion, testis cell suspension was filtered through 100 μm and 40 μm nylon mesh (BD Biosciences, San Jose, CA, USA), and centrifuged at 1500 rpm for 5 min at 4 °C. TECs were isolated by MACS with anti-CD31 antibody microbeads. To separate TEC-negative population excluding germ cells, CD31^−^ cells were then used for MACS with anti-Thy-1 microbeads and Thy-1^-^ testicular cells were collected, resulting in CD31^−^ Thy-1^−^ testicular cells. The 2D co-cultures were generated by plating 3.0 × 10^5^ cultured GFP^+^ SSCs on top of 200 μl of solid Matrigel mixture containing TEC or TEC-negative testicular cells in serum-free medium.

### Western blot analysis

Testes harvested from vehicle or busulfan-treated wild-type mice were homogenized in RIPA buffer and protein concentration quantified by the BioRad DC Protein Assay. Per sample, 25 µg of protein was separated by sodium dodecyl sulfate–polyacrylamide gel electrophoresis (SDS–PAGE), probed with anti-GDNF rabbit polyclonal antibody (1:500; Santa Cruz, Cat: 13147) or anti-cleaved Caspase 3 rabbit polyclonal antibody (1:1000; Cell Signaling, Cat: 9664) and detected by chemiluminescence (ECL, Amersham). Blots were stripped and re-probed with β-actin as a loading control. See Supplementary material for uncropped blots.

### Immunohistochemistry

Testes dissected from male mice were cryoprotected overnight in 20% (wt vol^−1^) sucrose and then frozen in OCT (Tissue-Tek). Testis sections (10 or 30 μM) were blocked (5% normal goat serum and 0.1% bovine serum albumin in 0.1% phosphate-buffered saline with Tween-20 (PBS-T)) for 1 h and washed in 0.1% PBS-T. Primary antibodies, anti-CD31 rat-pAb (1:50; BD science, Cat: 550274), anti-PLZF rabbit polyclonal Ab (1:50; Santa Cruz, Cat: 22839), anti-DDX4 rabbit pAb (1:200; Abcam, Cat: 13840), anti-Sox9 rabbit pAb (1:200; Millipore, Cat: ABE579), anti-GDNF rabbit polyclonal Ab (1:50; Santa Cruz, Cat: SC328), were diluted in blocking buffer and incubated for 2 h at room temperature (RT), then incubated with either anti-mouse Alexa 594 (1:1000, Thermo Fischer, Cat: A110323) or anti-rabbit Alexa 488 (1:1000, Thermo Fischer, Cat: A32723) at RT for 30 min. Sections were stained for 1 min to detect nuclei. Immunofluorescence images of testis regular sections were captured with AxioVision software (Zeiss) mounted on a Zeiss Imager M2 microscope or 30 μM thickness sections were captured *Z*-stack with Zeiss LSM 710 confocal, then all *Z*-stack images were reconstructed as a projection images by ImageJ (National Institutes of Health). Digital images were analyzed for the area and density of endothelial cell markers, germline stem cell markers and GDNF by counting 5 random 20× fields per testis section.

Mice were killed 5–6 weeks after busulfan treatment and tissues were fixed in paraformaldehyde overnight and then embedded in paraffin. Slides were cut in 5 μM sections. For antigen retrieval, the sections were baked at 60 °C for 60 min and subjected to antigen retrieval using DAKO target antigen retrieval solution (Dako, Carpinteria, CA) at 99 °C for 20 min. Sections were blocked in normal donkey serum and then incubated with primary antibody overnight. Secondary antibody to the appropriate species followed by amplification with streptavadin-horseradish peroxidase was used. Slides were stained with AEC^+^ substrate. Bright-field images were captured by Olympus BX51 (Olympus) with an AxioCam digital camera (Zeiss). A random section of testis was examined under 200× and the field searched for complete tubules seen in cross-section without evidence of obvious fixation artifact. After all cross-sectional areas were counted, the field was randomly moved and all cross-sectional tubules counted in that field. This was continuing until the 10–20 tubules were counted per slide.

### GDNF ELISA and mRNA

Testis, lung, or liver ECs were plated (10,000 cells ml^-1^) on 12 well dishes coated with 0.1% gelatin and cultured for 24 hours. Conditioned media was harvested at 24 and 48 hours after FGF-2 (2 ng ml^-1^, 20 ng ml^-1^ and 50 ng ml^-1^) treatment and analyzed by enzyme-linked immunosorbent assay (ELISA) for GDNF (Promega GDNF Emax immunoassay system, #G7621) following the manufacturer’s instruction. Statistics was analyzed using GraphPad Prism. For quantification of *Gdnf* mRNA, total RNA was isolated from the cells using Direct-zol RNA MiniPrep (Zymo Research) and reverse transcribed using High Capacity cDNA Reverse Transcription Kit (Applied Biosystems), according to the manufacturer’s instructions. All gene expression levels were normalized to *Gapdh* mRNA levels.

### Testicular cell proliferation

1 × 10^4^ total testicular cells were plated onto gelatin-coated 8-well LabTek chamber slides and cultured overnight. TEC-derived GDNF in the media was neutralized by the addition of 2 µg ml^−1^ of GDNF antibody or IgG1 at 4 ^o^C overnight followed by the addition of protein G at 4 ^o^C. After GDNF depletion was confirmed by GDNF ELISA, conditioned media were added to testicular cells for 24 h. Testicular cells were fixed with 4% paraformaldehyde followed by blocking in 5% normal goat serum and permeabilization in 0.1% bovine serum albumin in 0.1% PBS-T. Proliferation was detected by immunostaining with anti-Ki67 rabbit polyclonal-FITC Ab (1:50; Abcam) or isotype-matched control antibodies. Primary antibodies were added for 1 h. Cells were washed with PBS-T before the addition of anti-mouse-Alexa 594 (1:1000; Molecular Probes) for 30 min and protected from light. Nuclei were stained with 1% 4′,6-diamidino-2-phenylindole (DAPI) for 1 min. Cells were washed with PBS, mounted and imaged as described above. Ki67^+^ and SSC^+^ cells were counted in 10 random fields and the percent positive cells were analyzed by software prism (GraphPad Prism).

### Transplantation of GFP^+^ SSCs

Five to six -week-old male C57Bl/6 mice were treated with one dose of busulfan (45 mg kg^−1^, Sigma) by intraperitoneal (i.p.) injection to deplete SSCs. After 6 weeks, busulfan-treated C57Bl/6 mice were used as recipients. Because of their lack of endogenous spermatogenesis, W/Wv mice were used as recipients for transplantation experiments to produce offspring. To quantify donor-derived spermatogenesis, donor cells were transplanted into busulfan-treated C57Bl/6 mice. To determine whether donor cells were capable of producing offspring, donor cells were transplanted into 4–6-week-old W mutant mice. The recipients were anesthetized i.p. with 75 mg kg^−1^ ketamine and 0.5 mg kg^−1^ medetomidine and microinjected with either 10 μl of 3D Matrigel cultured GFP+Thy-1.2+SSC or GFP+testicular or lung endothelial cells (5 × 10^8^ cells per ml) isolated from C57Bl/6 GFP-ubiquitin transgenic mice into the testes of recipient mice. Approximately 10 μl (2.5 × 10^6^ cells per ml) of donor cells were transplanted into busulfan-treated C57Bl/6, resulting in approximately 80% filling of the seminiferous tubules. To conduct progeny generation transplantation, approximately 2 μl (70 × 10^6^ cells per ml) of donor cells were transplanted into W mutant mice through efferent ducts and then 1.5 μl (50 × 10^6^ cells per ml) of TECs were directly injected into inner space of testis. At 3 months after transplantation, recipient testes were analyzed for donor-derived spermatogenesis under fluorescent microscope. All animal procedures were performed according to the approved guidelines of the Animal Care and Use Committee of Chung-Ang University in accordance with the Guide for the Care and Use of Laboratory Animals of the National Institutes of Health (IACUC assurance no. 11–0038) and the University of Pennsylvania (Philadelphia, PA) Institutional Animal Care and Use Committee (IACUC protocol no. 804423).

### Mice

C57BL/6J mice were obtained from Jackson Laboratories. VE-cadherin-Cre-ER mice were provided by Ralf Adams. *Fgfr1*^fl/fl^ mice were generated as previously described^8^. All mice were used at 5–6 weeks of age. All animal experiments were performed under the guidelines set by the University of Pennsylvania IACUC and approved by the same committee. WBB6F1-W/Wv mutant mice (W mutant mice), obtained from the Jackson Laboratory (Bar Harbor, ME, USA), were used as recipients for transplantation.

### SSC-enriched cultures

The 2D co-cultures were generated by plating 2.7 × 10^5^ cultured GFP^+^ SSCs on STO feeder cells^28^ or on top of 200 μl of solid matrigel mixture containing TEC (BD Bioscience) in serum-free medium^28^ with exogenous GDNF 10 ng ml^−1^ (R&D System), GFRα1 75 ng ml^−1^ (R&D system) and FGF-2 1 ng ml^−1^ (BD Biosciences). Average GFP^+^ SSC colony area was measured and GFP^+^ SSC cell number were counted 8 days after plating. Each condition was performed in triplicate.

The 3D spheroid colonies were generated by isolation of Thy-1.2^+^ GSCs from the testes of 6–8-day-old mice. Next, 50,000 freshly isolated Thy-1.2^+^ germ stem cells were mixed with 25,000 TECs or LuECs in a 2:1 mixture of Matrigel and Dulbecco’s modified Eagle's medium/F12 (Gibco) containing 10% FBS, 2 mmol l^−1^ l-glutamine, 100U L^−1^ penicillin–streptomycin and 1 ml of ITS universal (BD Bioscience) and plated into 8-well LabTek chamber slides (Thermo Scientific). Cultures were incubated at 37 ^o^C for 40 min to solidify followed by the addition of germ stem cell media to the top of the solid Matrigel mixture. Cultured Thy-1.2^+^ SSC spheroid colony numbers and the average diameter of colonies were measured on the indicated days after plating.

### Transplantation of TECs into Ts65Dn mice

Nine-week-old Ts65Dn male mice were anesthetized with Avertin (250 mg kg^−1^) by i.p. injection for surgical cell transplantation. At transplantation, 10 μl (0.5 × 10^8^ cells per ml) of TECs from C57BL/6 wild-type GFP-ubiquitin transgenic mice were microinjected into the testis of Ts65Dn mice. After transplantation, the surface tubules of the testes were filled about 50%, and Trypan blue was used to examine cell death.

### iPSC differentiation

To generate iPSC lines, either human fibroblasts/stromal cells were transduced with pMXs-based retroviral supernatant with human OCT4, SOX2, KLF4, or MYC as previously described^30^, or mononuclear cells were infected with pHage2-CMV-RTTA-W and pHage-Tet-hSTEMMCA-loxP virus as previously described^31^. All cells were cultured on 0.1% gelatin-coated dishes in human endothelial cell medium (Lonza; EGM^®^-MV Bulletkit) with 50 ng ml^−1^ additional VEGF. Then, 500,000 disaggregated single embryonic bodies from human iPSCs were cultured for 48 h. Non-adherent cells were gently removed and adherent cells were cultured for 1–2 passages. Cells at 80–90% confluence were dissociated with enzyme free cell dissociation solution (Millipore) for 30 min and isolated with a human CD34 microbead kit (Miltenyi Biotec) following the manufacturer’s instruction. Isolated CD34^+^ cells were cultured until cells were confluent. CD34^+^ cells were selected with human CD31 microbead kit (Miltenyi Biotec) and CD34^+^CD31^+^ cells were characterized by Ac-LDL uptake, immunostaining with CD31, VEGFR2 and VE- cadherin, and matrigel tube formation assay.

### ChIP

ECs were transfected with caNFATc1^55^ and cultured in endothelial cell growth medium. Cells were washed with PBS, crosslinked for 10 min in 1% formaldehyde, quenched with 0.125 M glycine, washed with PBS, then lysed (10 mM Tris pH 8.0, 10 mM NaCl, 0.2% NP-40, protease inhibitors, H_2_O) and cytoplasmic contents removed. Nuclei were then lysed (50 mM Tris pH 8.0, 10 mM EDTA, 1% SDS, protease inhibitors, H_2_O) and sonicated. Samples were precleared with protein G and 50 µg of mouse IgG for 2 h at 4 ^o^C. Samples were centrifuged, pellets discarded and supernatant incubated overnight with antibody bound beads (10 µg antibody and protein G slurry). Antibodies used were anti-NFATc1 (Sc-7294; Santa Cruz) and mouse IgG. Crosslinking was reversed, DNA eluted, and qPCR performed for the region between −1000 and −830 on the *Egr1* promoter, using primers F: 5’-ACCTAGAACAATCAGGGTTCCGCA and R: 5’-AGTGTCCCAAGAACCAGTAGCCAA. The negative control primers cover the region from −548 to −420 on the *Egr-1* promoter, and the sequences are primers F: 5’-AGACCTTATTTGGGCAGCGCCTTA and R: 5’-GCCACTGCTGCTGTTCCAATACTA.

### GFP^+^ Thy-1.2^+^ germline stem cell and TEC transplantation

The 5–6-week-old male C57Bl/6 mice were treated with one dose of busulfan (45 mg kg^−1^, Sigma) by i.p. injection to deplete SSC^19^. After 6 weeks, busulfan-treated mice were anesthetized with 2–4% isoflurane and microinjected with either 10 μl of 3D Matrigel cultured GFP^+^Thy-1.2^+^ SSC (5 × 10^6^ cells per ml) or GFP^+^ testicular or lung endothelial cells (5 × 10^8^ cells per ml) isolated from C57Bl/6 GFP-ubiquitin transgenic mice into the testes of recipient mice. All animal procedures were performed according to the approved guidelines of the Animal Care and Use Committee of Chung-Ang University in accordance with the Guide for the Care and Use of Laboratory Animals of the National Institutes of Health (IACUC assurance no. 11–0038) and the University of Pennsylvania (Philadelphia, PA) IACUC (IACUC protocol no. 804423).

### Statistical analysis

Prism (GraphPad) was used for graphing and statistical analysis of data. Statistical significance was determined by *T*-test (two-tailed unpaired) and two-way analysis of variance (ANOVA) test between two groups.

## Electronic supplementary material

Supplementary Information

Description of Additional Supplementary Files

Supplementary Movie A

Supplementary Movie B

## Data Availability

The authors declare that the data supporting the findings of this study are available within the article and its Supplementary Information files or are available upon requests to the authors.
