# Supplementary material for: Testicular endothelial cells are a critical population in the germline stem cell niche
Source: Nat Commun. 2018 Oct 22;9:4379. doi: 10.1038/s41467-018-06881-z (PMC6197186; doi:10.1038/s41467-018-06881-z)

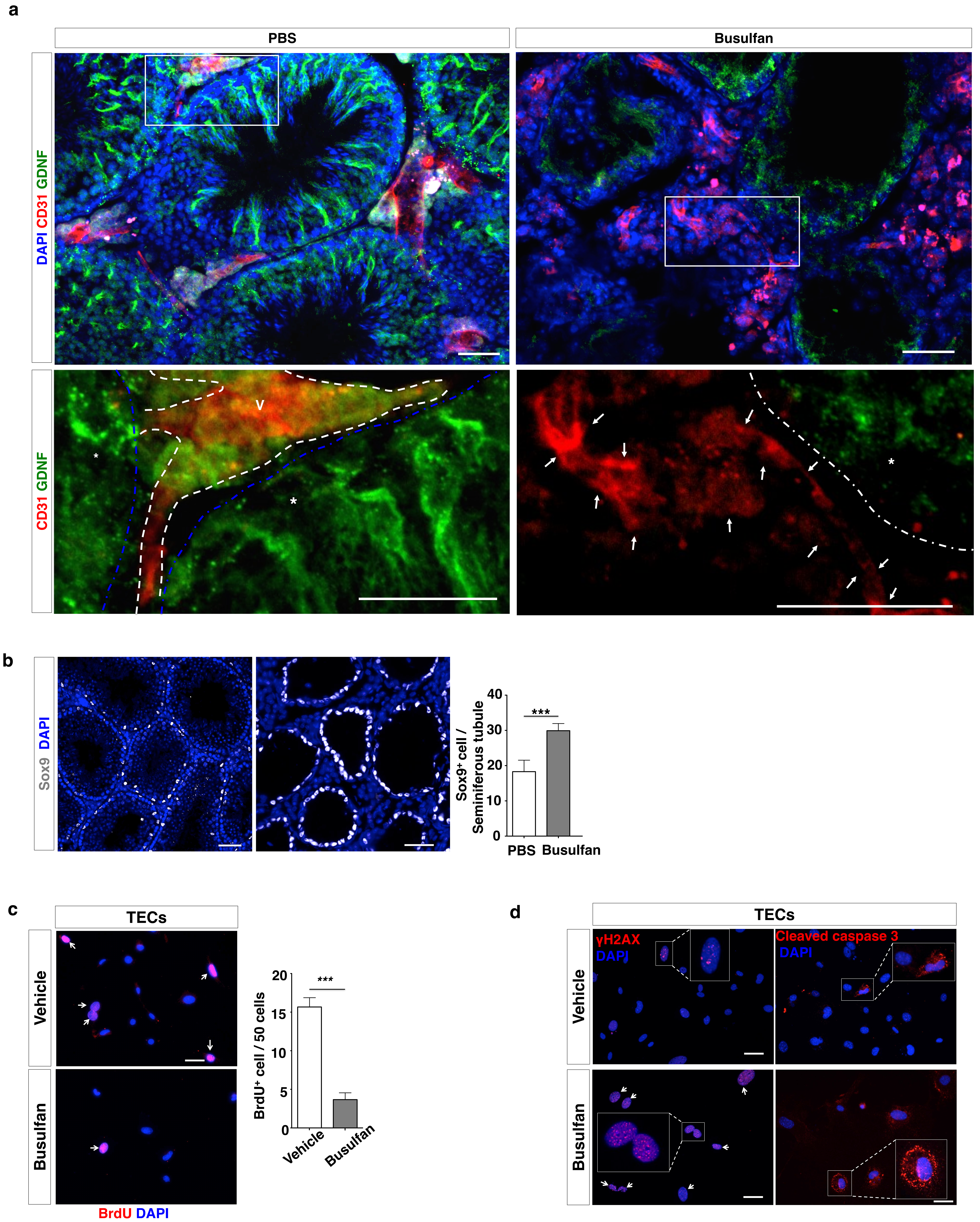

Supplementary Figure 1. GDNF expression in testis sections from wild-type mice after busulfan treatment. a Representative immunofluorescence images of testis sections from wild-type (WT) mice 5 weeks after PBS or busulfan treatment (45 mg kg^{-1}). Sections were immunostained for CD31 and for GDNF expression. * indicates seminiferous tubules; arrows, damaged microvessels; V and dotted lines, vessels. Bar = 50 μM. **b** Immunofluorescence staining for Sox9 to identify Sertoli cells on testes sections from wild type (WT) mice 5 weeks after PBS or busulfan treatment. Quantification of Sox9^{+} cells per seminiferous tubule is shown on the right. n=4-5, Sox9^{+} cells in seminiferous tubule were quantified by counting 5 random 20x fields per testis section. Two tailed unpaired T-test, ***P<0.001. **c** TEC proliferation *in vitro* was examined by BrdU uptake after vehicle or busulfan treatment. Quantification of BrdU^{+} cells is shown on the right. Data is representative of 1 of 3 independent experiments. **d** Representative immunofluorescence images for γ-H2AX (red) or cleaved caspase 3 (red) expression in TECs 96 hours after vehicle or busulfan treatment *in vitro*. Nuclei were stained with DAPI (blue).

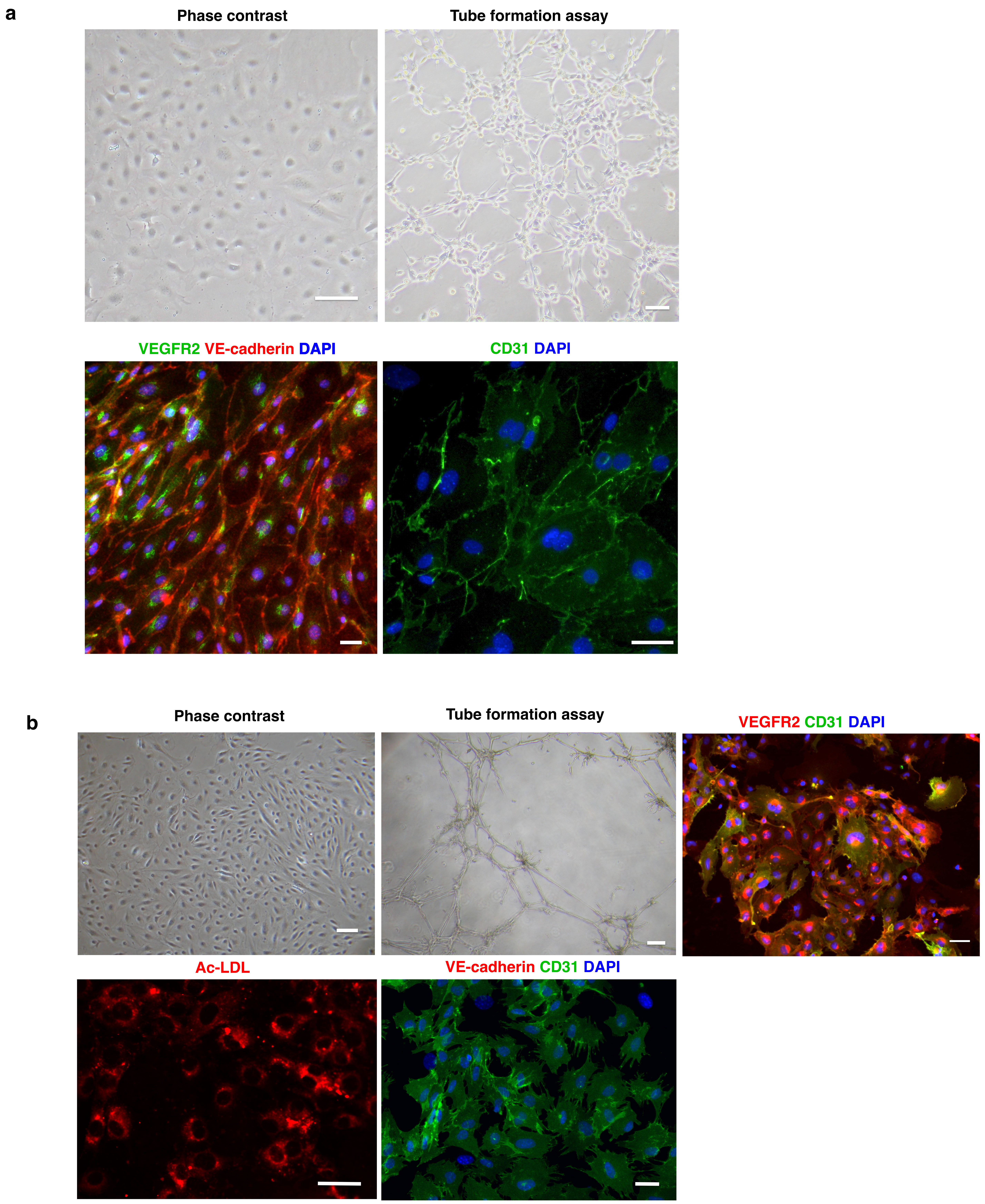

Supplementary Figure 2. Characterization of testicular endothelial cells. Primary mouse (**a**) testicular and (**b**) lung endothelial cells were isolated from WT C57Bl/6 male mice and endothelial identity examined by phase contrast, tube formation on Matrigel, Acetylated-LDL (Ac-LDL) uptake and immunostaining with the endothelial markers VEGFR2, VE-cadherin and CD31. Bar = 50 μM.

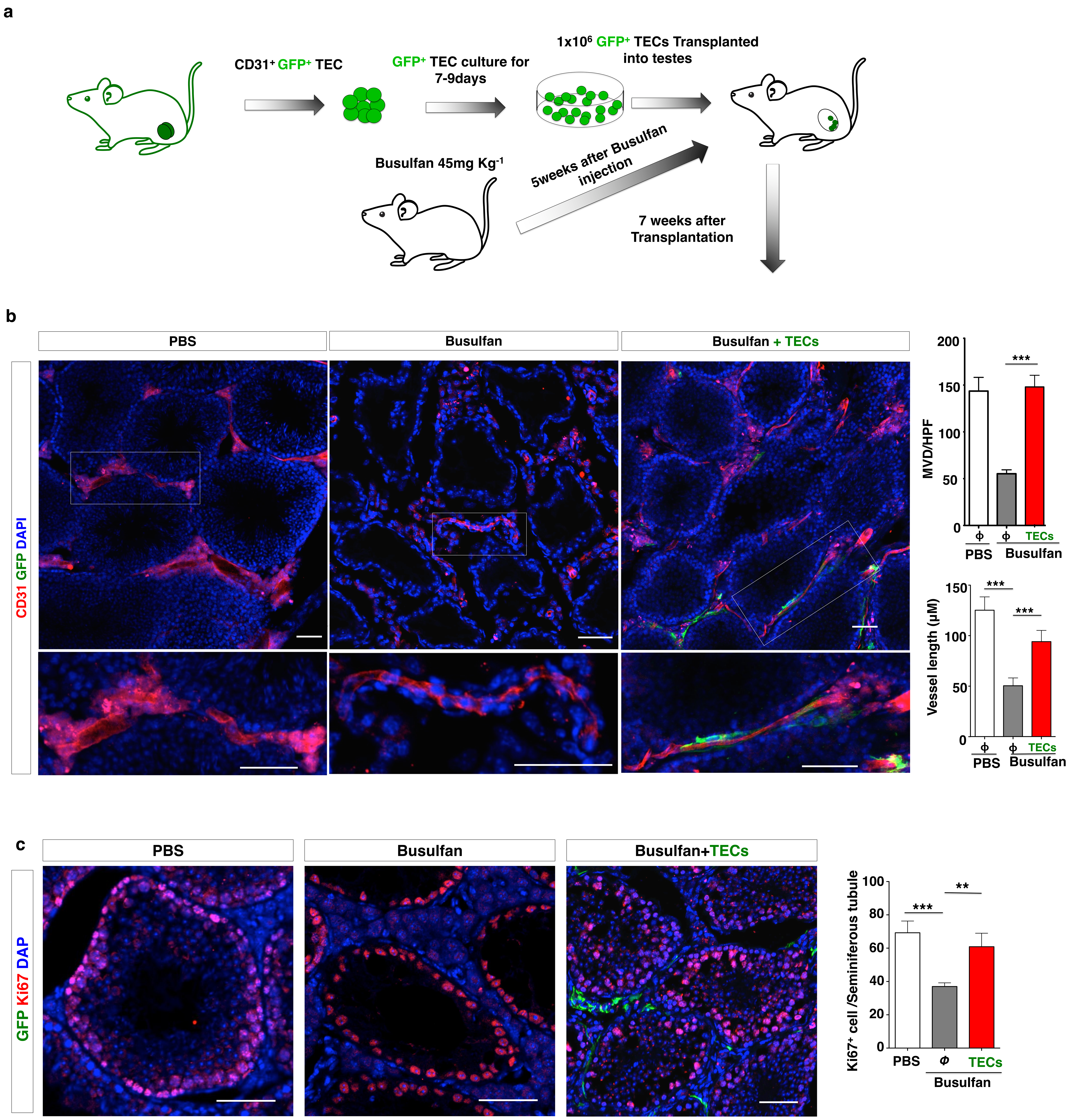

Supplementary Figure 3. Busulfan affects TECs. a. Schematic and timeline for TEC transplantation into busulfan-treated mice.
b Immunofluorescence images of testis sections from wild-type mice after vehicle (PBS), busulfan or busulfan plus GFP+ TEC transplantation. Sections were immunostained for CD31 (red), GFP (green), and DAPI (blue). Quantification of microvessel density (MVD) (n=9 random high power field (HPF) images) and vessel length (n=30 vessels in random HFP images) is shown on the right, 8-9 mice per group were examined.
c Immunofluorescence images for the proliferation marker Ki67 in testis sections from WT mice after treatment with PBS, busulfan or busulfan + GFP+ TECs transplantation. Ki67+ cells per seminiferous tubule are quantified on the right, n=3~4. Data are presented as the mean ± s.e.m. Bar = 50 μM. Two tailed unpaired T-test, **P*<0.05. ***P*<0.01, ****P*<0.001. Two tailed unpaired T-test.

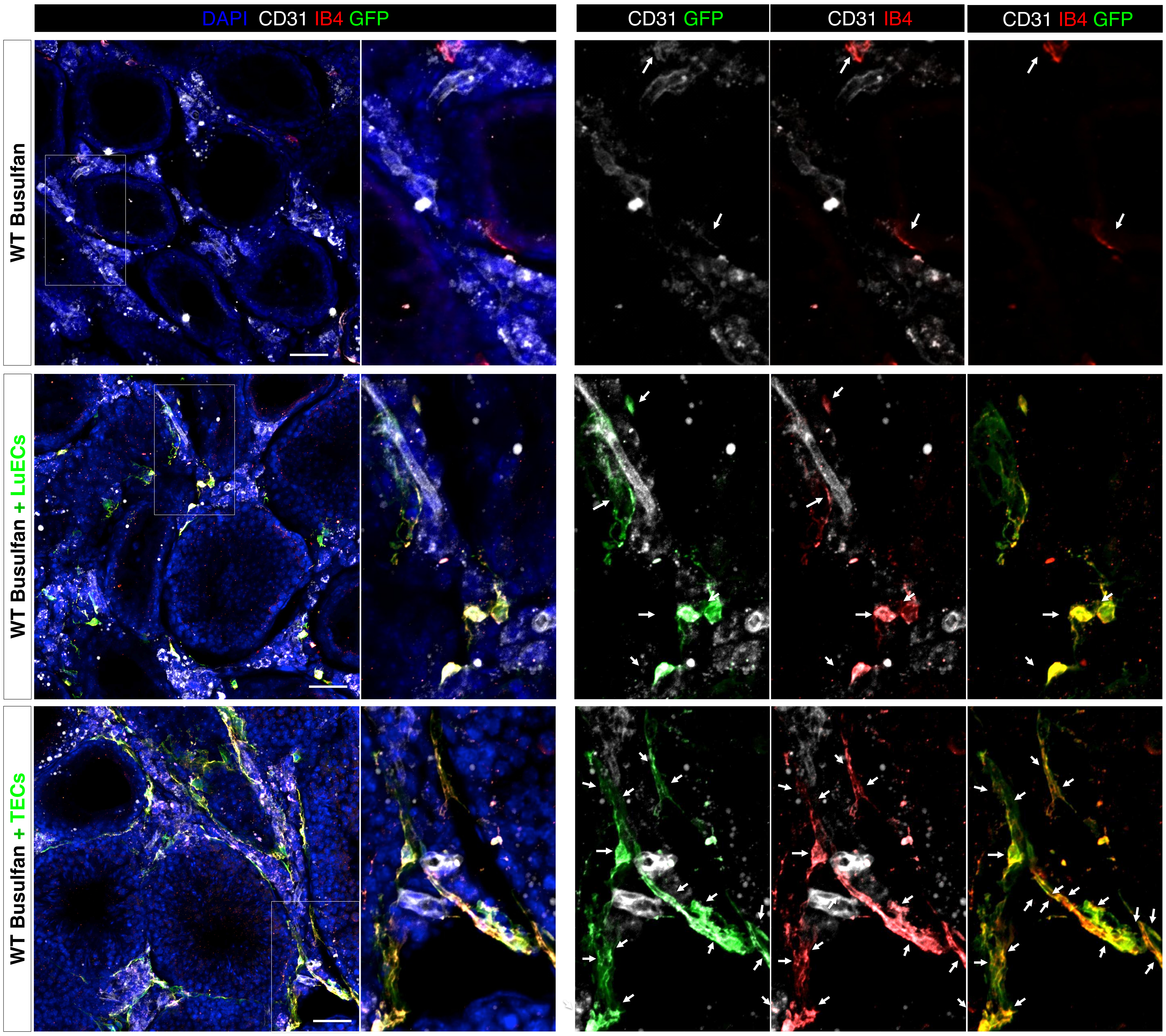

Supplementary Figure 4. Transplanted TECs are functional. Immunofluorescence images of testis sections after Isolectin B4-Alexa 594 injection into the testes of WT mice treated with busulfan or busulfan + GFP+ TECs or busulfan + GFP+ lung endothelial cells (LuECs). Sections were co-immunostained with the EC marker CD31 and GFP. Arrows indicate co-localization of CD31, isolectin B4 and GFP. Bar = 50 μM.

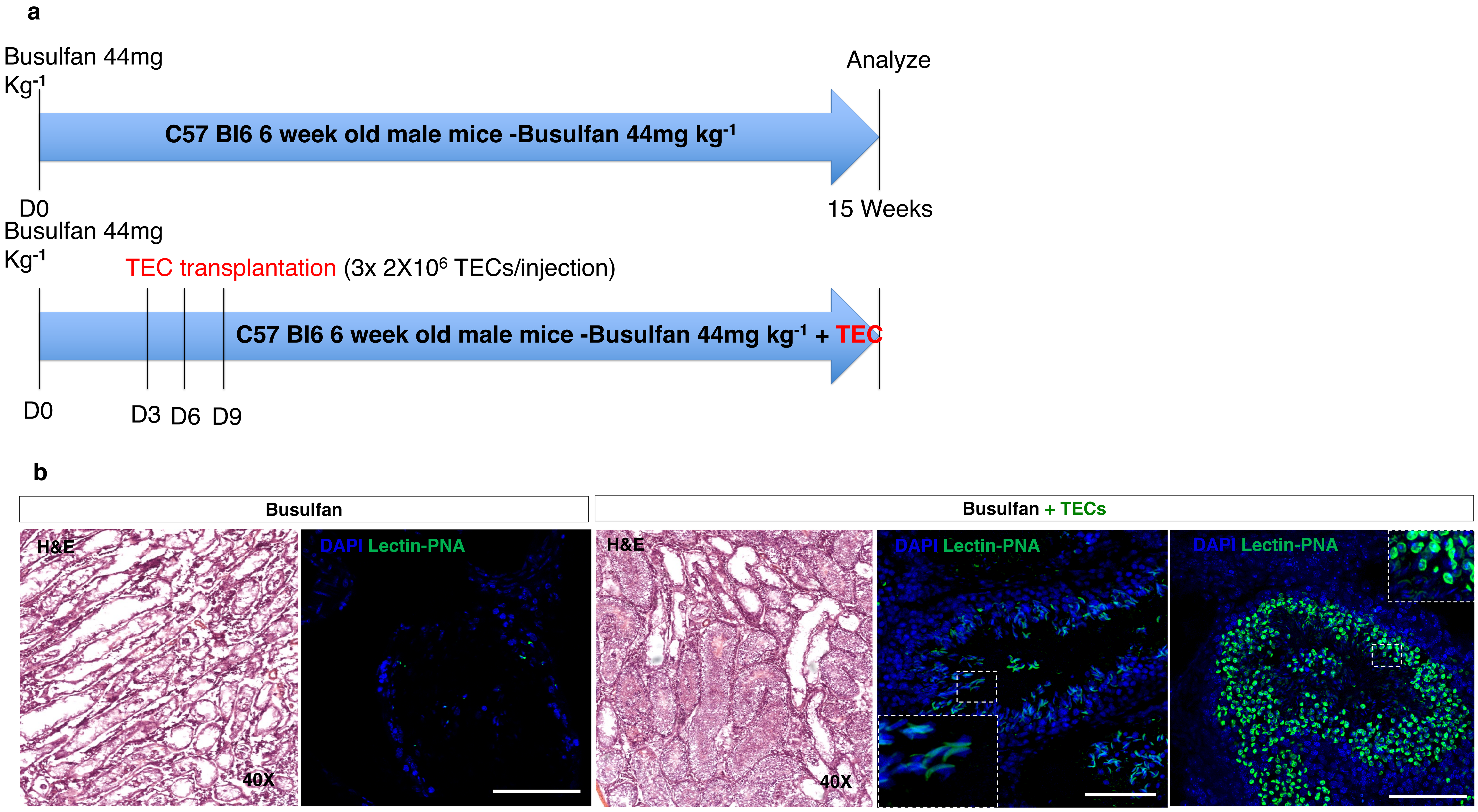

Supplementary Figure 5. TEC transplantation protects SSCs from busulfan-mediated cell death. a Schematic and time line of TEC transplantation into busulfan injected mice. **b** Representative images of lectin PNA immufluoresence and histology from sections of testes from busulfan injected mice with or without TEC transplantation. Bar = 50 µM.

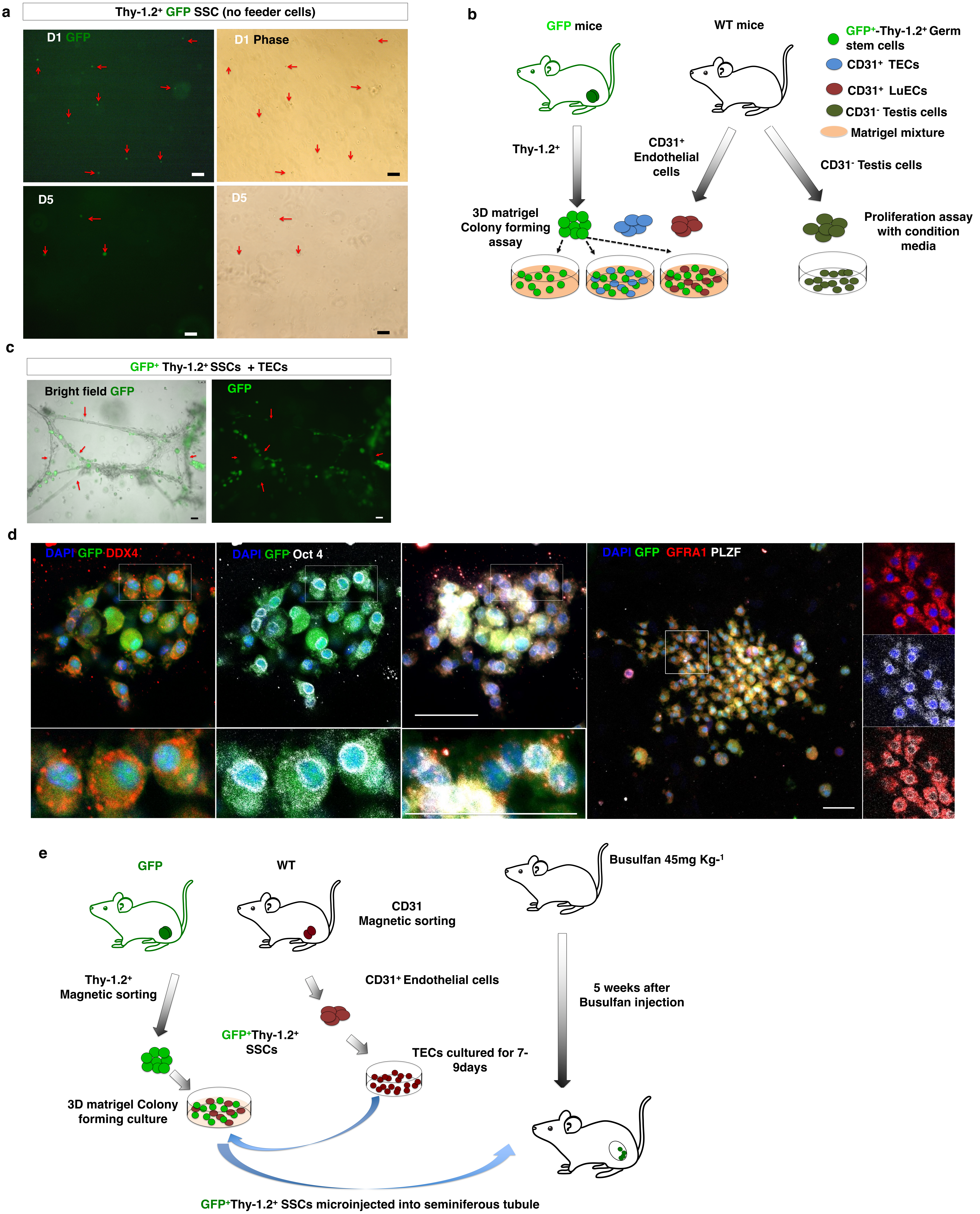

Supplementary Figure 6. Testicular endothelial cells can support proliferation of germ stem cells containing SSCs *in vitro*. a Representative GFP or phase contrast images of GFP⁺Thy-1.2⁺ SSCs cultured in the absence of TECs. Red arrows indicate the few remaining SSCs 1 or 5 days (D1 or D5) after seeding. **b** Schematic representation of 3D-Matrigel colony formation and proliferation of Thy-1.2⁺ SSC *in vitro*. **c** Representative bright field images merged with a GFP image of GFP⁺Thy-1.2⁺ SSCs localizing to TEC tubes after seeding. Representative GFP image shows formation of 3D colonies (red arrows) along TEC tubes. **d** GFP⁺Thy-1.2⁺ SSC colonies cultured with TECs were immunostained with the germ stem cell markers DDX4, Oct4, GFRA1 and PLZF 14 days after seeding **e** Schematic representation of transplantation procedure of 3D co-cultures of GFP⁺Thy-1.2⁺ SSCs with TECs into the testes of mice 5 weeks after busulfan treatment. Bar = 50 μM.

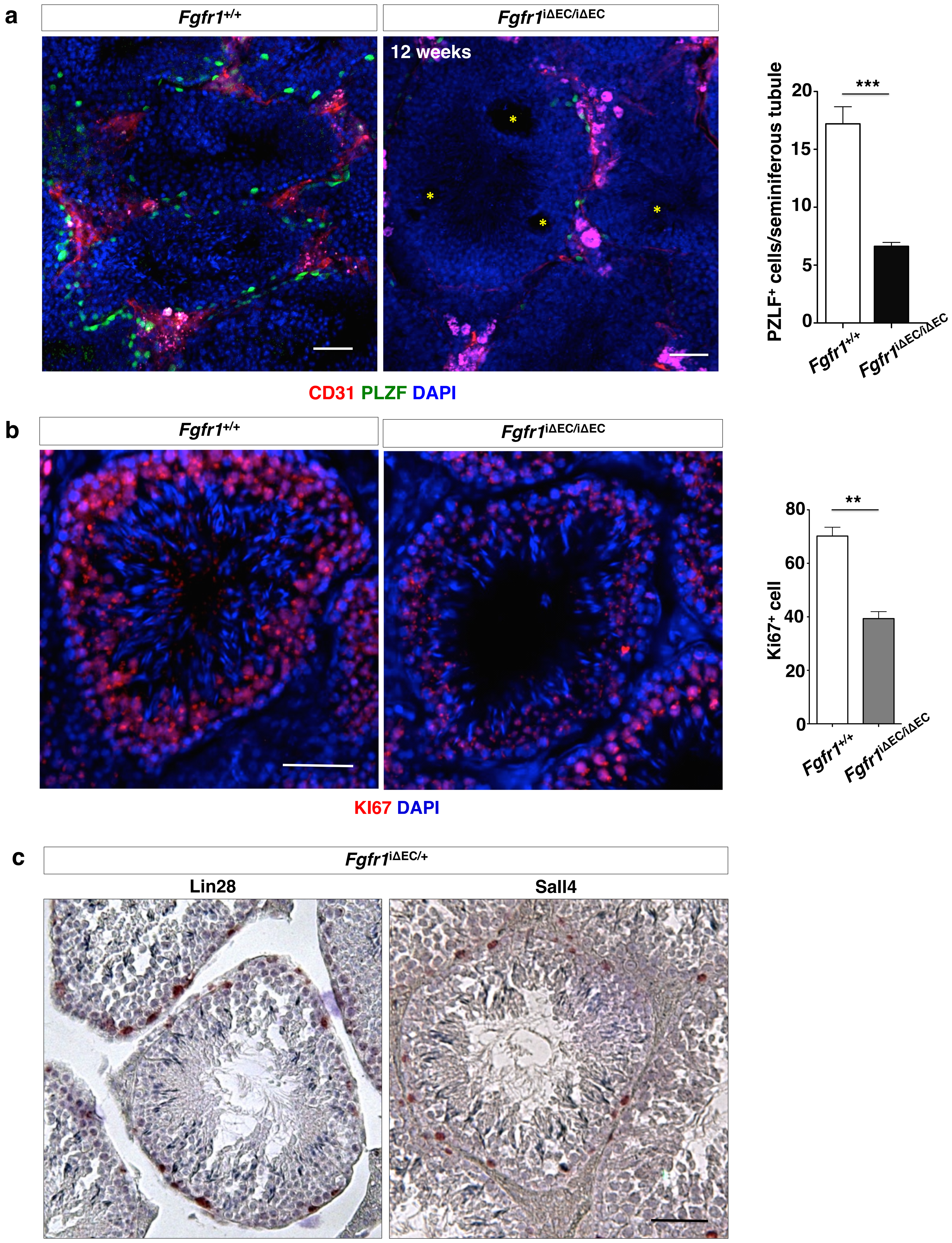

Supplementary Figure 7. FGFR1 deletion in TECs attenuates SSC proliferation and numbers. a Immunofluorescence images of testes sections from control (*Fgfr1*$^{+/+}$, n=3) or endothelial-specific deletion of *Fgfr1* (*Fgfr1*$^{i\Delta EC/i\Delta EC}$, n=3) mice after tamoxifen treatment. Sections were immunostained for CD31 (red) and the germ stem cell marker PLZF (white). PLZF^{+} cells were quantified on the right (n=15 random HPF images per group). ***P<0.001. **b** Immunofluorescence images for the proliferation marker Ki67 (red) on testes from *Fgfr1*$^{+/+}$ (n=3) or *Fgfr1*$^{i\Delta EC/i\Delta EC}$ **(**n=4**)** mice. Ki67^{+} cells in seminiferous tubules are quantified on the right (n=assessed seminiferous tubule number; *Fgfr1*$^{+/+}$,n=60; *Fgfr1*$^{i\Delta EC/i\Delta EC}$, n=74.) **P<0.001. **c** Immunohistochemical image for *Sall4* and *Lin28* on testes sections from *Fgfr1*$^{i\Delta EC/+}$ mice (n=4) 5 weeks after busulfan treatment (10ng Kg^{-1}). Data are presented as the mean ± s.e.m. Two tailed unpaired T-test.

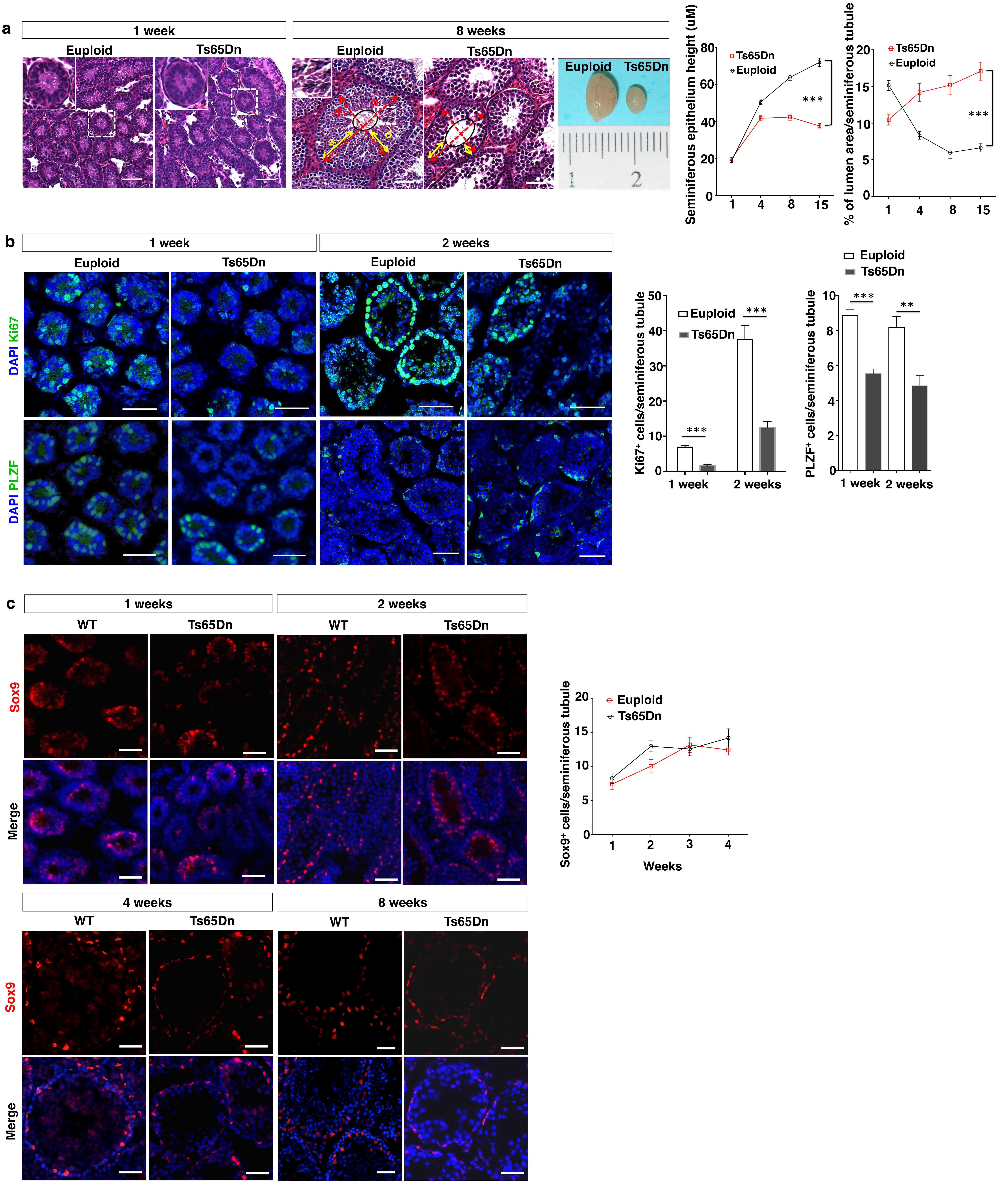

Supplementary Figure 8. The Ts65Dn mouse model of Down Syndrome exhibits defects in spermatogenesis and SSC maintenance. **a** Images from H&E stained sections from euploid (n=5) and Ts6Dn (n=6) mice on indicated age. Magnified image (white rectangular box) from euploid shows sperm. Quantification of the height of the seminiferous epithelium ((a+b)/2) and % of lumen area in seminiferous tubule ((axb)/(a'xb')x100) is indicated on the right. Two way Anova test. **b** Immunofluorescence images of the proliferation marker Ki67 and the stem cell marker PLZF on testes section from euploid (n=4) and Ts65Dn (n=4) mice at the indicated ages. Ki67⁺ and PLZF⁺ cells are quantified on the right. Two tailed unpaired T-test. **c** Immunofluorescence images of testes section from euploid (n=4) and Ts65Dn (n=4) mice probed for the sertoli cell marker Sox9 at the indicated ages. Sox9⁺ cells are quantified on the right. All data are presented as the mean ± s.e.m. Bar = 50 μM. $^{**}P<0.01$, $^{***}P<0.001$.

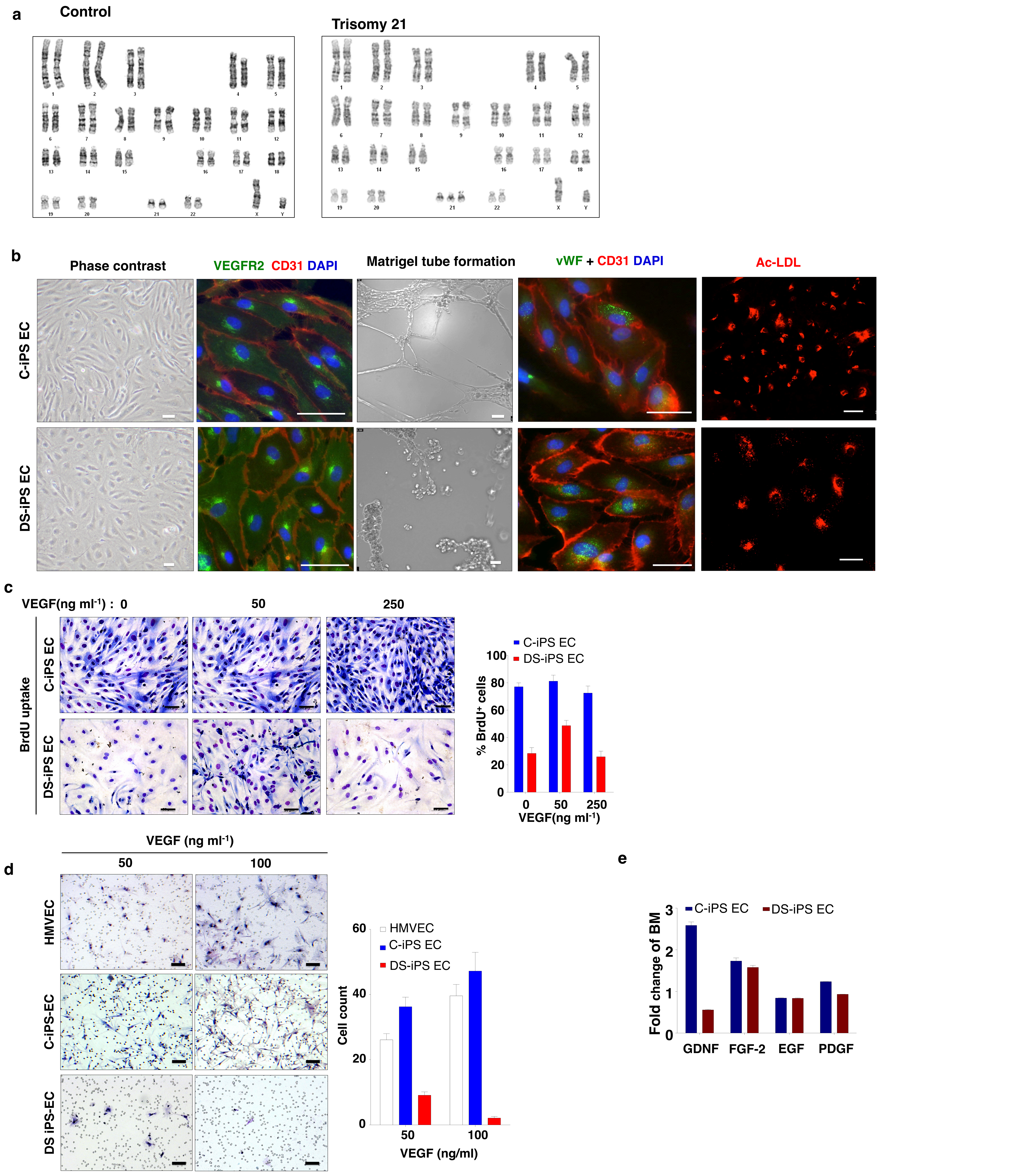

Supplementary Figure 9. Characterization of induced pluripotent stem cell (iPSC)-derived ECs from a Down syndrome individual.
a Karyotype of control and Down syndrome (Trisomy 21) iPSCs used to generate endothelium. **b** Endothelial cell identity of control (C-iPS) and Down syndrome (DS-iPS) iPSC-derived ECs was examined by phase contrast, immunostaining with VEGFR2, von Willebrand factor (vWF) and CD31, capillary tube formation on Matrigel and by acetylated-LDL (Ac-LDL) uptake. **c** Representative images of BrdU uptake by C-iPS EC and DS-iPS EC at the indicated VEGF concentrations for 2 days. BrdU uptake is quantified on the right. **d** Images from transwell migration with human microvascular endothelial cell lines (HMVEC)ca, C-iPS EC and DS-iPS EC in the presence of the indicated VEGF concentrations with quantification on the right. (E). Quantification of angiogenesis-related proteins expressed in the secretome of C-iPS EC and DS-iPS EC by antibody array. All data are presented as the mean ± s.e.m. Bar = 50 µM.

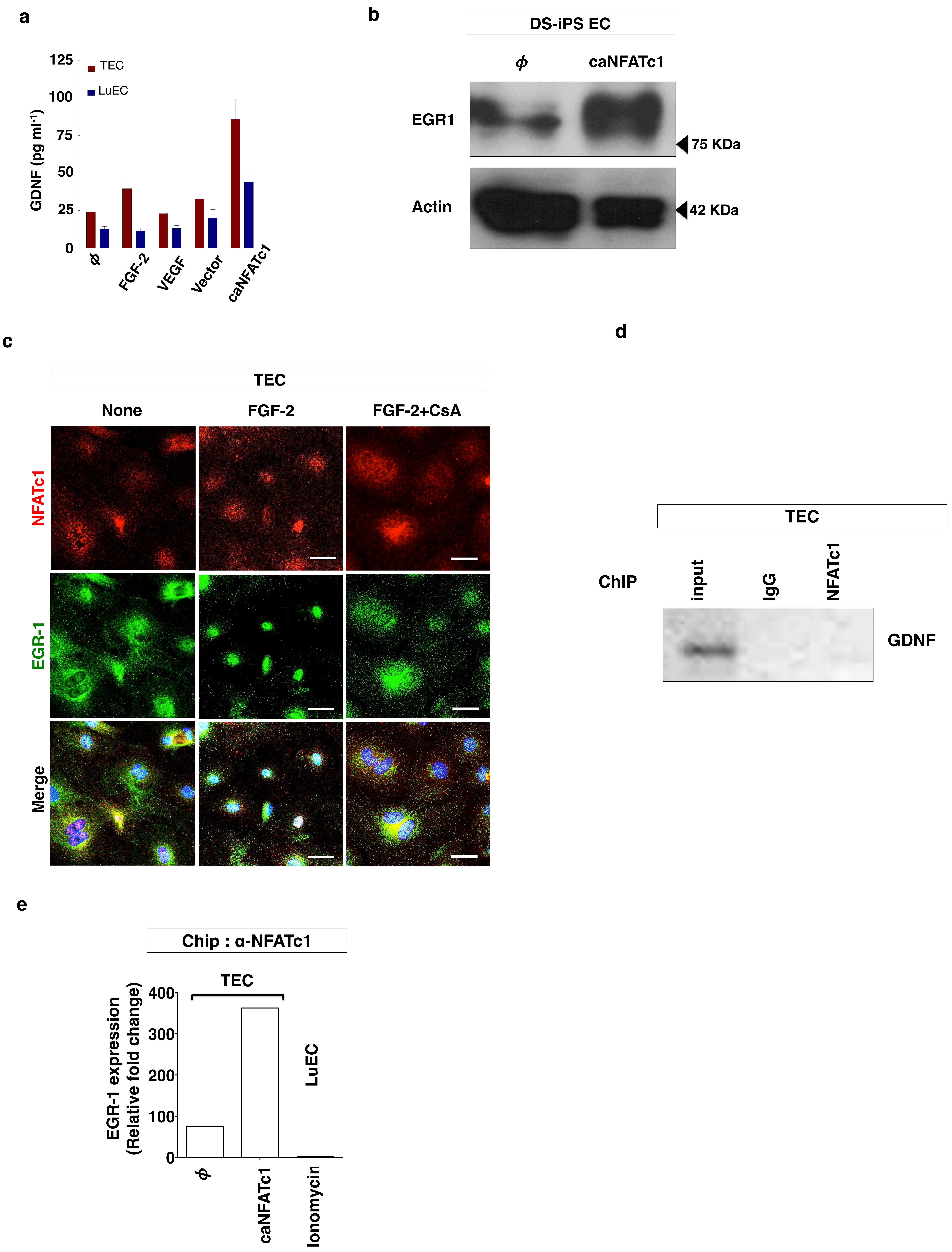

Supplementary Figure 10. FGF2 promotes EGR-1 production through the CaN-NFATc1 axis in TECs. a Quantification of GDNF levels by ELISA in conditioned media from TECs after FGF-2, VEGF treatment, expression of a constitutively active NFAT mutant (caNFATc1) or an empty vector control, n=3 replicates. Data are presented as the mean ± s.e.m. **b** Western blot of EGR1 expression in DS-iPS EC after expression of caNFATc1. **c** Immunofluorescence images of NFATc1 and EGR-1 localization in TEC after FGF-2 treatment with and without CsA. . Bar = 50 μM. **d** Chromatin immunoprecipitations (ChIP) of TECs with anti-NFATc1 mAb. NFATc1 was immunoprecipitated and DNA probed by PCR for NFATc1 consensus sites on the *Gdnf* promoter. IgG pulldown was used as a control. **e** *Egr-1* expression was quantified by PCR after NFATc1 ChIP of TEC before (untreated) or after expression caNFATc1. *Egr-1* expression was quantified after NFATc1 ChIP of LuEC after ionomycin treatment.

a

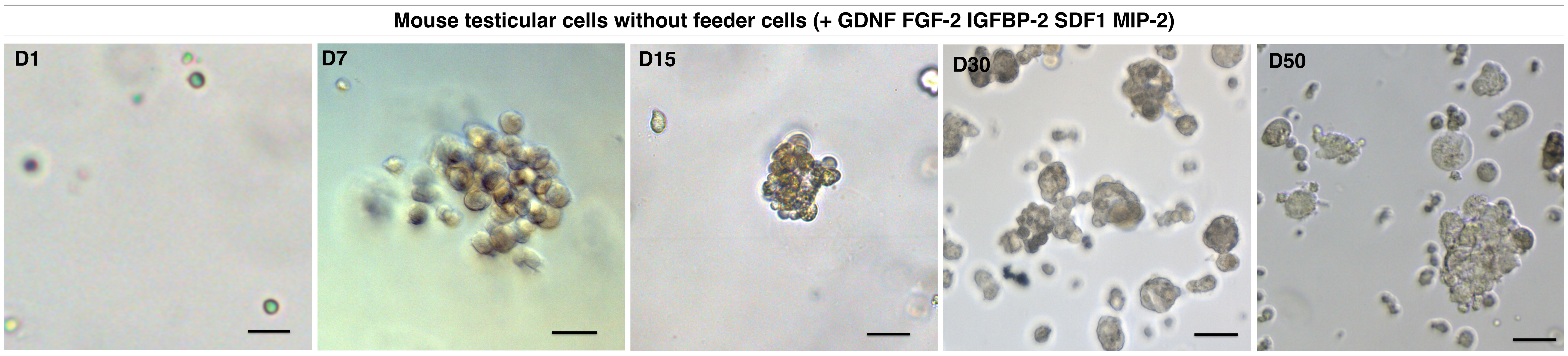

b

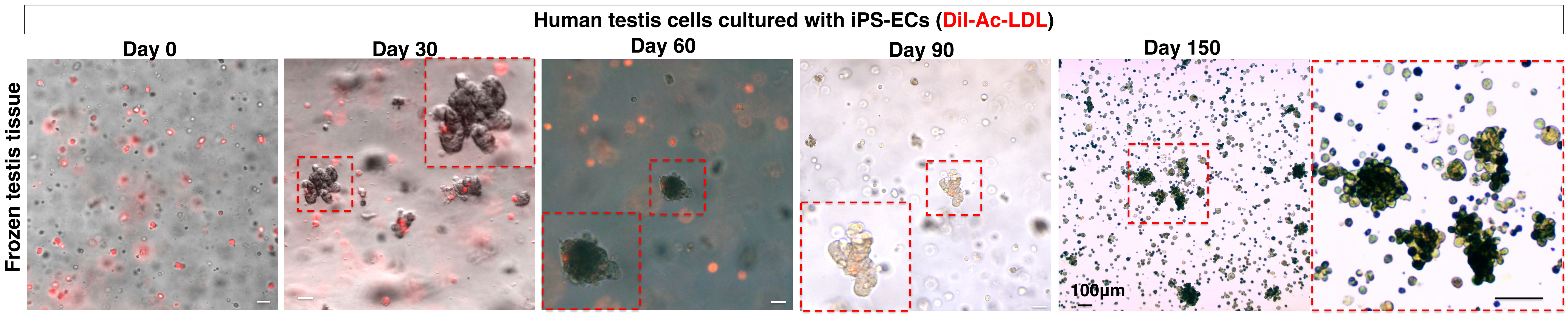

Supplementary Figure 11. Human and mouse SSCs can be expanded in the long term culture. Representative bright field images of (**a**) mouse SSC colonies in feeder free cultures in media containing GNDF, FGF-2, IGFBP-2, SDF-1 and MIP-2 and (**b**) human testis cells cultured with iPS ECs after Dil-Ac-LDL (red) uptake on the indicated days. Bar = 50 µM.

Figure 1c

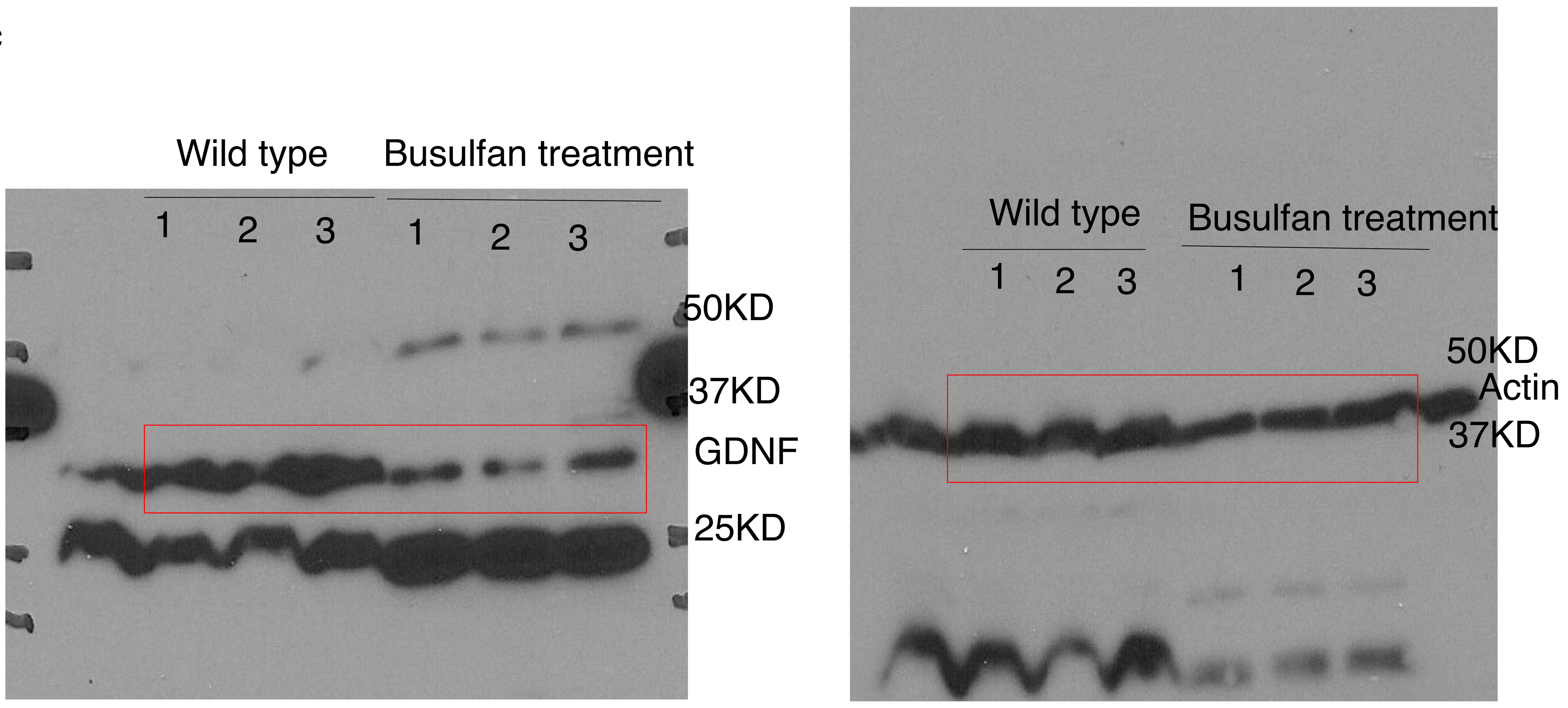

Figure 4e

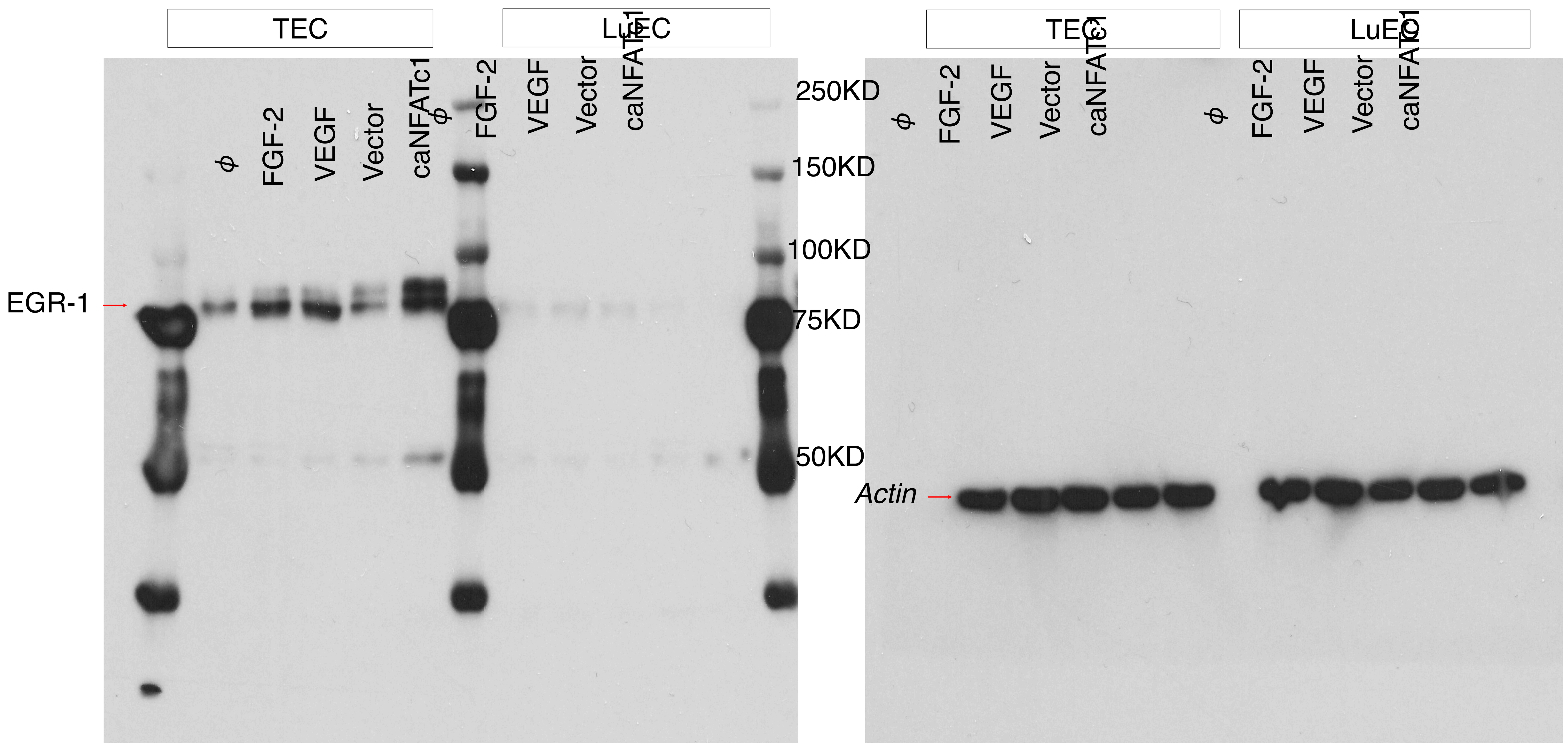

Figure 4f.

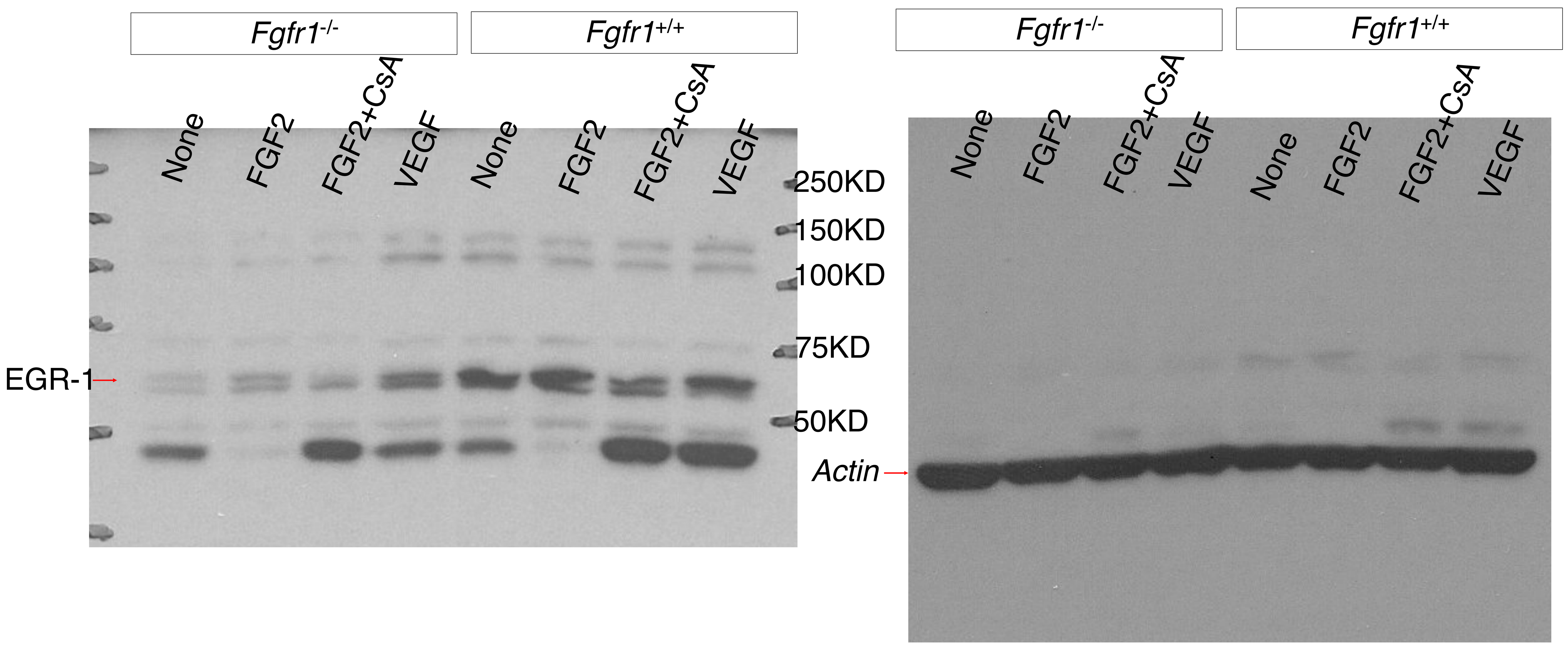

Figure 4h.

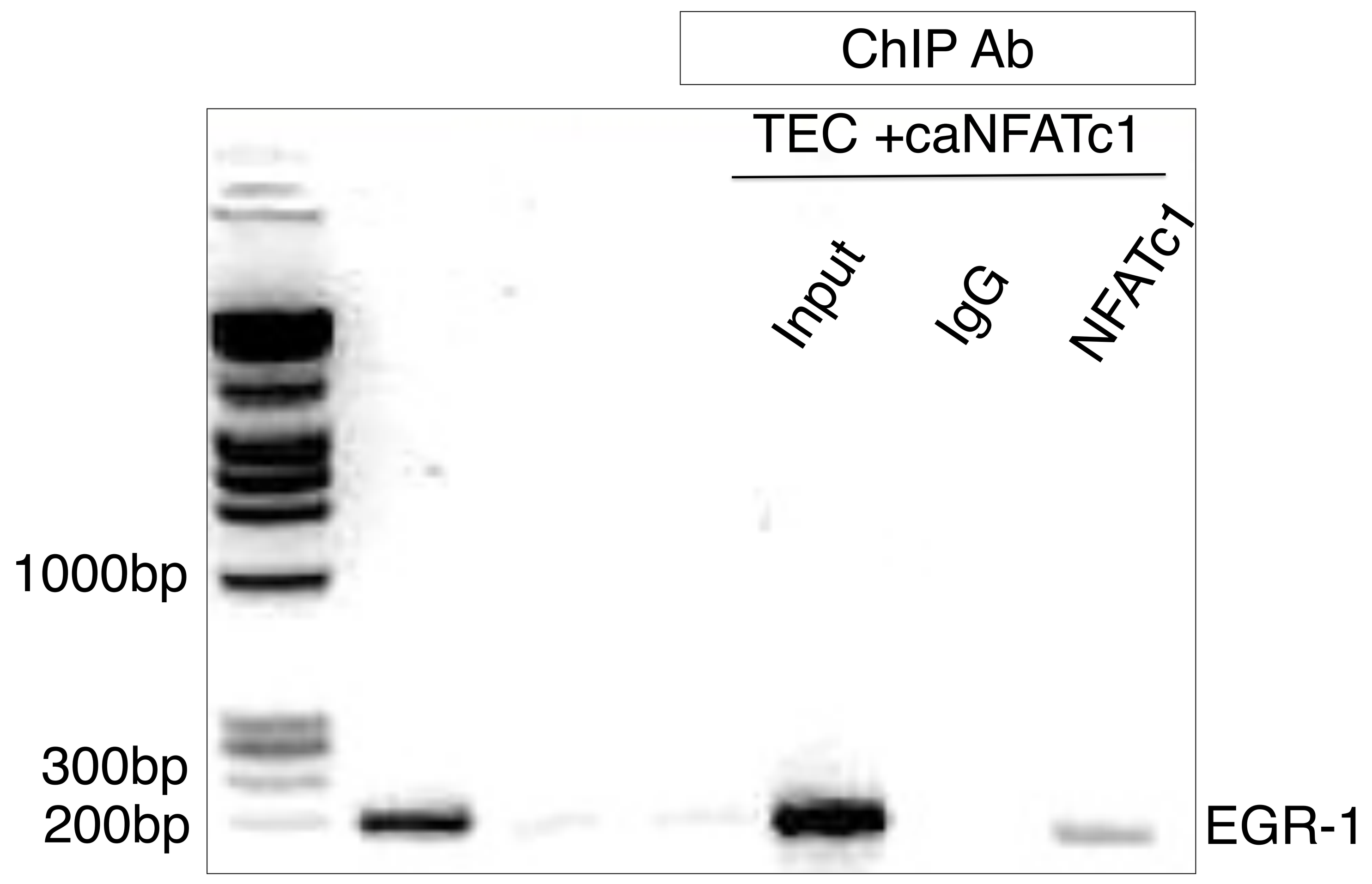

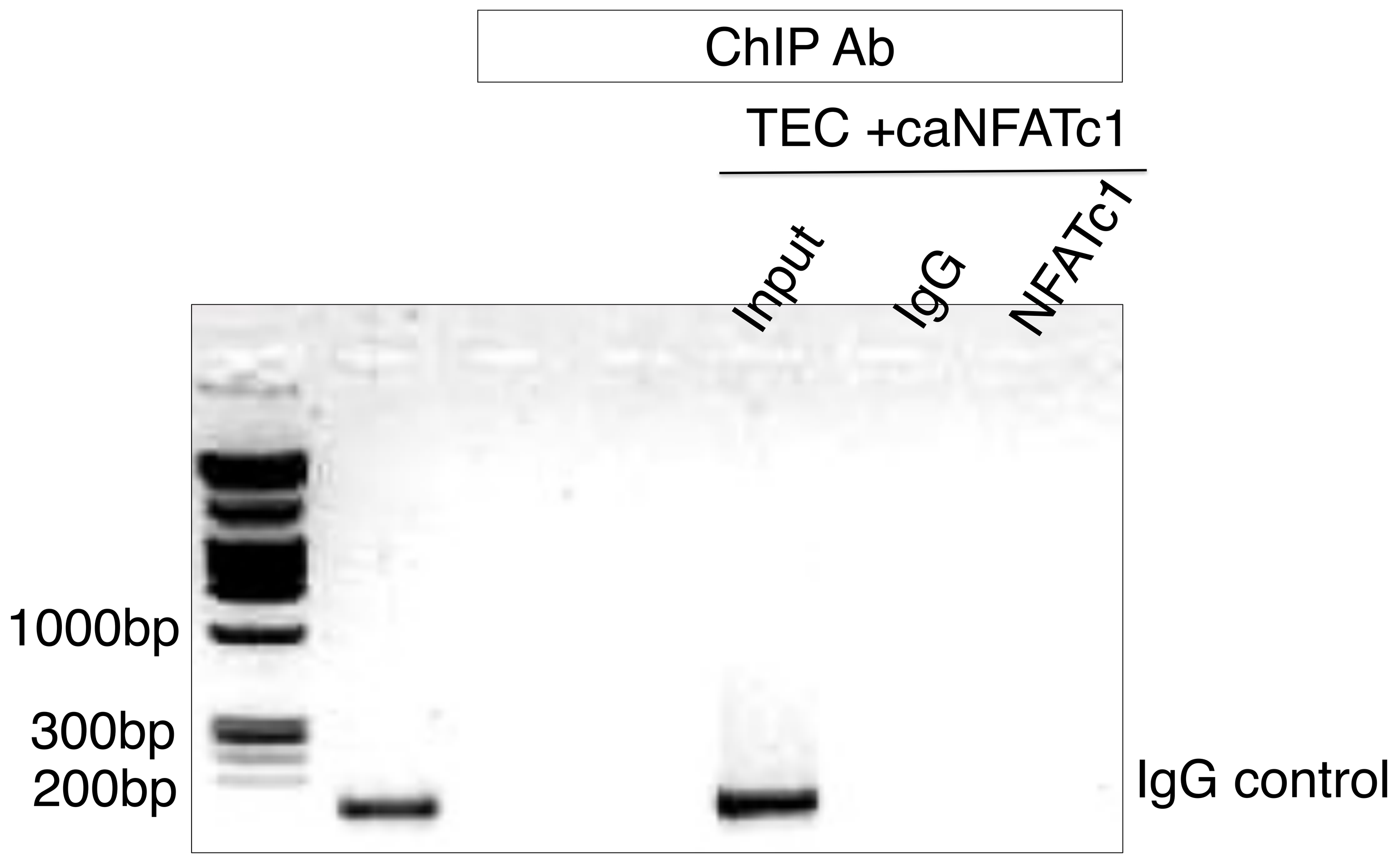

Supplementary Figure 9b.

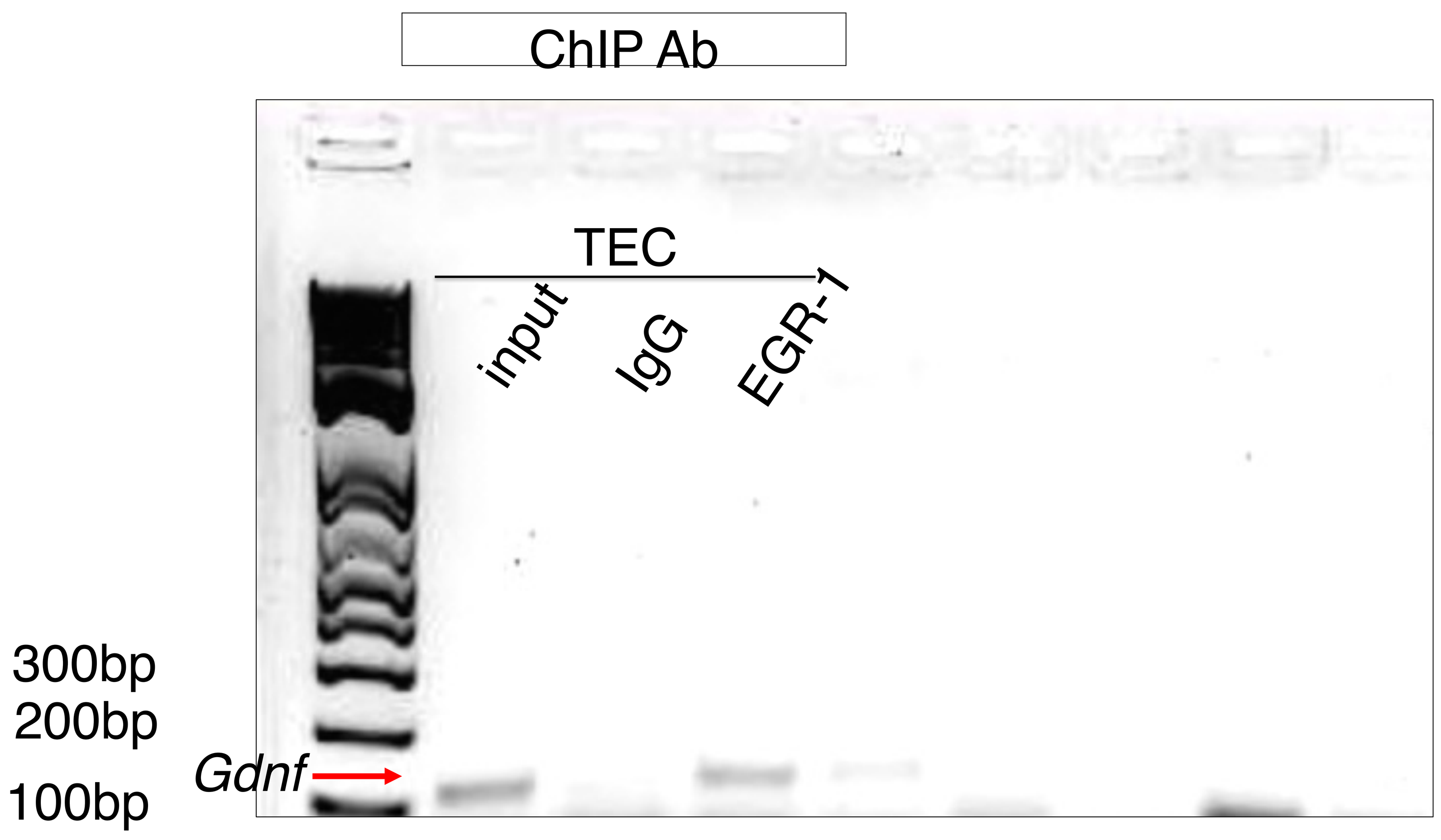

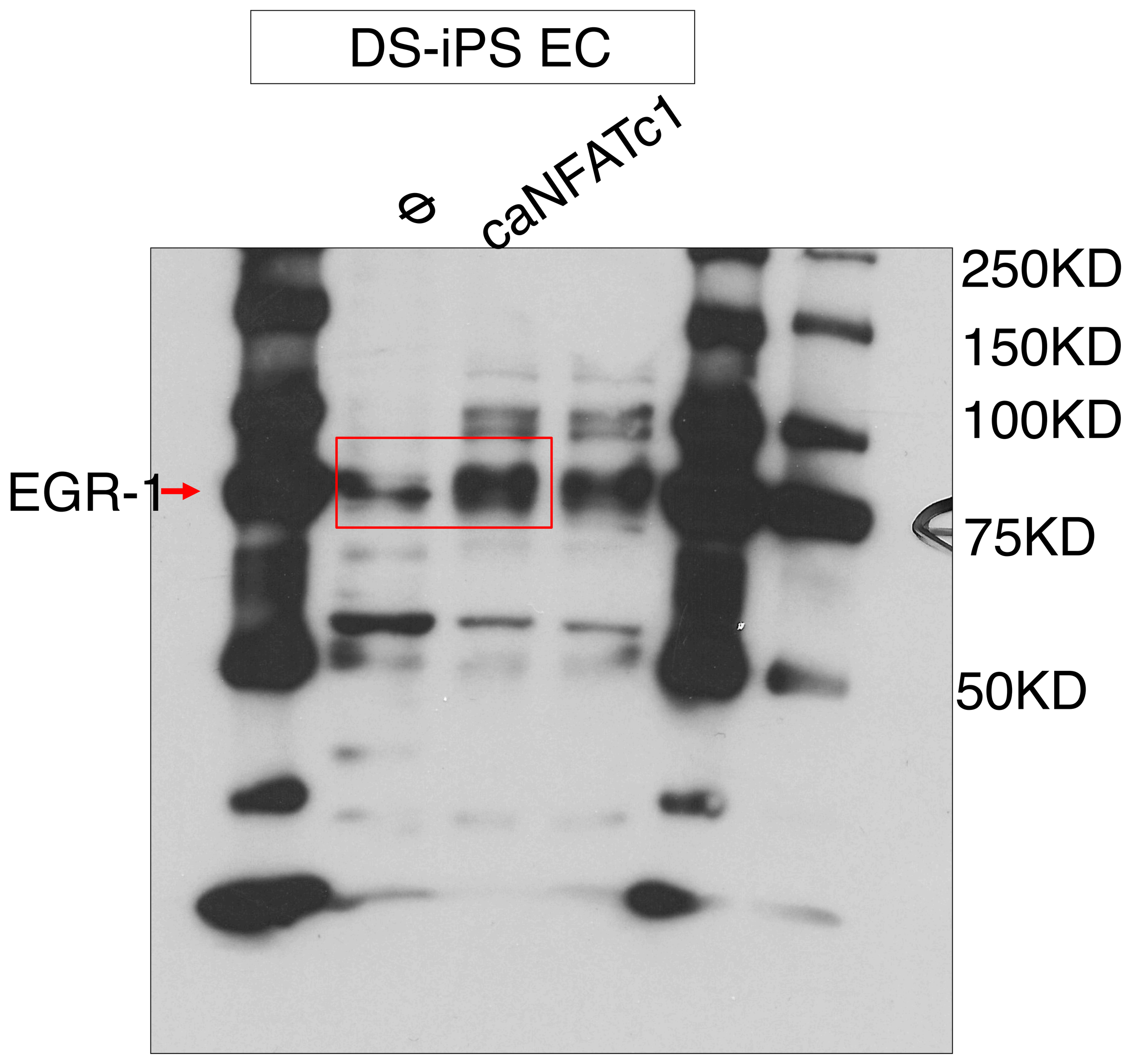

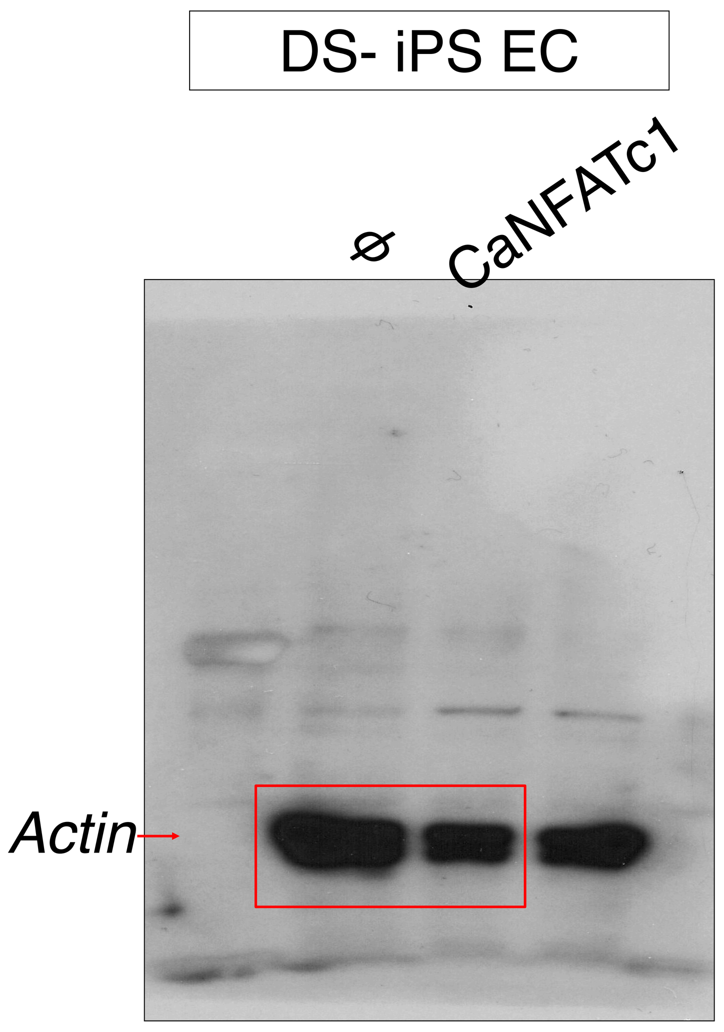

Supplementary Figure 9d.

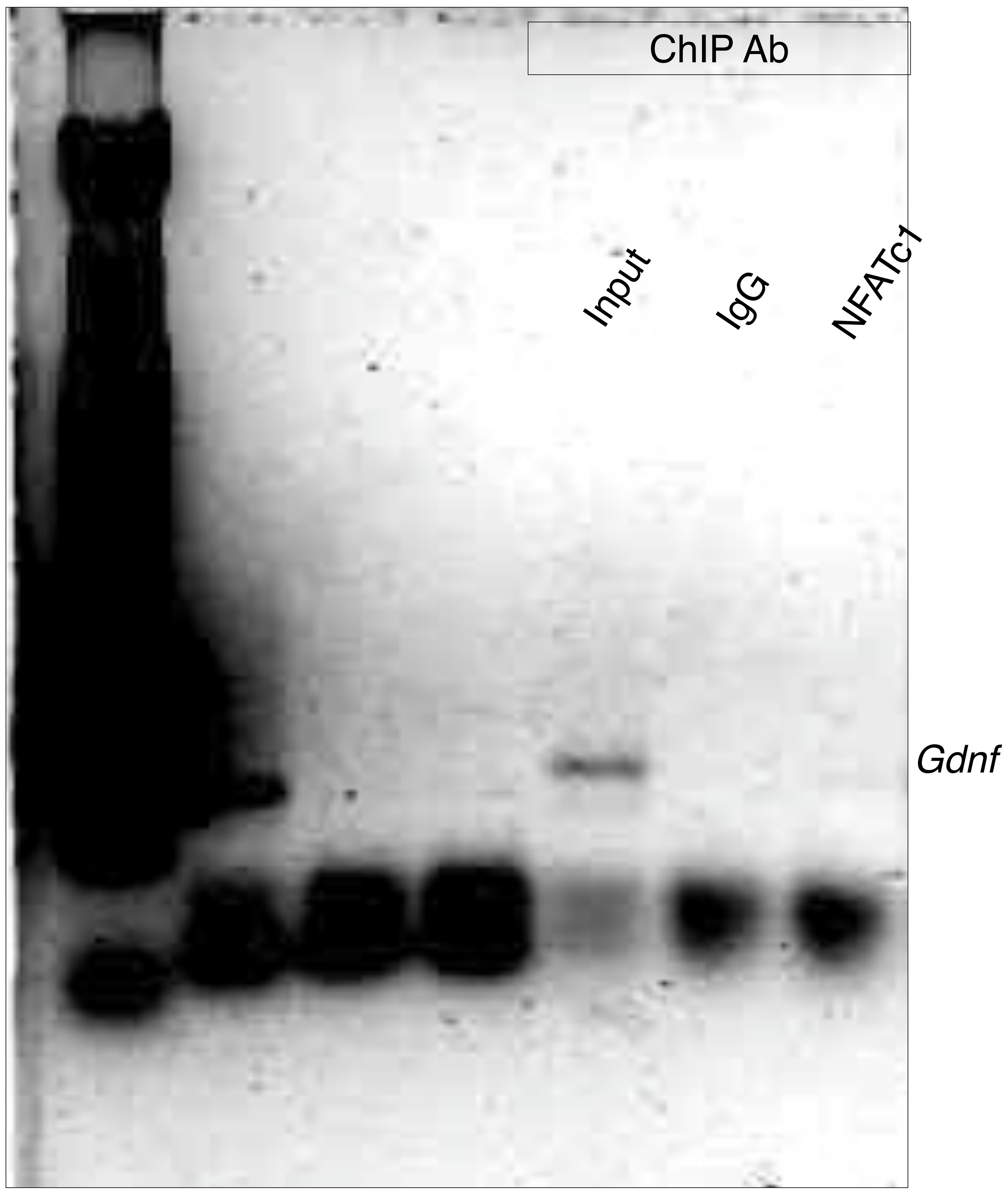

Supplement: Supplementary file 1 — Supplementary Information [file 41467_2018_6881_MOESM1_ESM.pdf]
