# Supplementary material for: Testicular endothelial cells are a critical population in the germline stem cell niche
Source: Nat Commun. 2018 Oct 22;9:4379. doi: 10.1038/s41467-018-06881-z (PMC6197186; doi:10.1038/s41467-018-06881-z)
Supplement: Supplementary file 2 — Description of Additional Supplementary Files [file 41467_2018_6881_MOESM2_ESM.pdf]

Description of Additional Supplementary Files

File Name: Supplementary Movie A
Description: Defects in 2D capillary tube formation on Matrigel by DS-iPS ECs.

File Name: Supplementary Movie B
Description: 2D capillary tube formation by C-iPS-ECs on Matrigel.
